# Discovery of a Novel MHC Class I Lineage in Teleost Fish which Shows Unprecedented Levels of Ectodomain Deterioration while Possessing an Impressive Cytoplasmic Tail Motif

**DOI:** 10.3390/cells8091056

**Published:** 2019-09-09

**Authors:** Unni Grimholt, Kentaro Tsukamoto, Keiichiro Hashimoto, Johannes M. Dijkstra

**Affiliations:** 1 Fish Health Research Group, Norwegian Veterinary Institute, Ullevaalsveien 68, 0454 Oslo, Norway; 2 Institute for Comprehensive Medical Science, Fujita Health University, Toyoake, Aichi 470-1192, Japan (K.T.) (K.H.)

**Keywords:** major histocompatibility complex, MHC, evolution, nonclassical, fish

## Abstract

A unique new nonclassical MHC class I lineage was found in Teleostei (teleosts, modern bony fish, e.g., zebrafish) and Holostei (a group of primitive bony fish, e.g., spotted gar), which was designated “H” (from “hexa”) for being the sixth lineage discovered in teleosts. A high level of divergence of the teleost sequences explains why the lineage was not recognized previously. The spotted gar H molecule possesses the three MHC class I consensus extracellular domains α1, α2, and α3. However, throughout teleost H molecules, the α3 domain was lost and the α1 domains showed features of deterioration. In fishes of the two closely related teleost orders Characiformes (e.g., Mexican tetra) and Siluriformes (e.g., channel catfish), the H ectodomain deterioration proceeded furthest, with H molecules of some fishes apparently having lost the entire α1 or α2 domain plus additional stretches within the remaining other (α1 or α2) domain. Despite these dramatic ectodomain changes, teleost H sequences possess rather large, unique, well-conserved tyrosine-containing cytoplasmic tail motifs, which suggests an important role in intracellular signaling. To our knowledge, this is the first description of a group of MHC class I molecules in which, judging from the sequence conservation pattern, the cytoplasmic tail is expected to have a more important conserved function than the ectodomain.

## 1. Introduction

### 1.1. The Structure and Function of Classical MHC-I

Classical MHC class I (MHC-I) molecules consist of extracellular α1, α2, and α3 domains, plus a connecting peptide (CP)/transmembrane (TM)/Cytoplasmic (CY) region. Such a “heavy chain” molecule forms a complex together with a single domain molecule β_2_-microglobulin (β_2_-m) and a peptide ligand, and this pMHC-I complex is presented at the surface of cells for screening by CD8^+^ T cells [1,2]. This system helps CD8^+^ T cells to detect virus-infected and cancerous cells, which can then be eliminated.

The α3 and β_2_-m domains are typical immunoglobulin superfamily (IgSF) structures, while the α1 and α2 domains form a unique structure of two anti-parallel helical structures on top of a β-sheet, which, in the case of classical MHC-I, forms a groove in which peptides of ~9 amino acids length can be bound [1]. Classical MHC molecules are renown for extensive polymorphism, which resides mostly in the peptide-binding domains and affects the sets of peptides presented by different allelic MHC molecules [3,4]. This polymorphism is believed to increase the resistance of a population against pathogens. Throughout jawed vertebrates (Gnathostomata), polymorphic sequences of classical MHC-I can be found [5], and for teleost fish, the ability of such molecules to bind β_2_-m and peptide ligand was proven and their ability to stimulate cytotoxic CD8^+^ T cells suggested by a variety of data ([6,7,8], reviewed in [9]).

### 1.2. Classical and Nonclassical MHC-I Can Show Differences in Domain Organization

At various times during evolution, classical MHC-I genes duplicated and new copies diverged into “nonclassical” MHC-I genes [10,11]. Depending on the respective molecule, nonclassical MHC-I molecules have retained similarity more or less with classical MHC-I and perform a wide variety of functions within and outside the immune system [12]. The evolutionary younger nonclassical MHC-I molecules especially tend to share features with the classical molecules, such as peptide binding, whereas many of the more ancient nonclassical lineages exhibit more diverged functions and properties [10,12]. The number of molecular domains can also differ from the classical MHC-I situation. For example, ZAG (zinc-α2-glycoprotein, alias AZGP1; [13]) is a soluble mammalian nonclassical MHC-I molecule which consists of α1, α2, and α3 domains, which does not bind β_2_-m and can bind fatty acids [14,15]. Another example is represented by EPCR (endothelial protein C receptor, alias PROCR; [16]) which is a nonclassical MHC-I molecule found in birds, reptiles, and mammals (reviewed in [10]), and which is a transmembrane molecule that promotes protein C activation (reviewed in [17]) and possesses only α1 and α2 ectodomains that form a hydrophobic groove that can bind hydrophobic molecules [18]. EPCR molecules belong to the same nonclassical MHC-I lineage as CD1 molecules [10,16], which are also found in mammals [19], birds, and reptiles (reviewed in [10]), although CD1 are transmembrane molecules that retained the α1, α2, and α3 ectodomain organization and β_2_-m binding ability of classical MHC-I. Like EPCR, CD1 molecules have a groove for binding hydrophobic molecules [20], but in the case of CD1, they present these to T cells (reviewed in [21]). Other nonclassical MHC-I transmembrane molecules in mammals without an α3 domain and without a β_2_-m partner are members of the RAET/ULBP (retinoic acid early transcripts/UL16 binding proteins) family [22]. RAET/ULBP molecules tend to have closed grooves, are not involved in presentation of small ligands, and can interact with NKG2D receptors on natural killer (NK) cells (reviewed in [12]); while some members of this family have transmembrane domains, others are associated with the membrane by means of a GPI anchor (reviewed in [23]). Although the evidence is somewhat thin [10], it has been proposed that the RAET/ULBP family forms a phylogenetic group together with the MIC/MILL family of NKG2D binding nonclassical MHC-I molecules which typically do possess an α3 domain [24]. Therefore, besides the EPCR/CD1 situation, the RAET/ULBP/MIC/MILL group may provide another example of related molecules with and without an α3 domain.

### 1.3. MHC-I in Teleost Fish and the Target of the Present Study

In teleost fish, there are currently five described MHC class I lineages denoted U, Z, S, L, and P, and all essentially contain transmembrane heavy chain molecules with the canonical three extracellular domains organization ([25,26,27,28,29]; reviewed in [30]), although at least at the genetic level, some variation in the number of domains can be found (e.g., [30,31,32]). Defined by polymorphism, expression pattern, and expectations for peptide binding ability, all identified teleost classical MHC-I genes belong to the U lineage, while the U lineage also contains nonclassical MHC-I genes (reviewed in [30]). Among teleost MHC-I, only classical U has been well studied at the genetic, structural, and functional levels (reviewed in [9]), whereas for Z, S, L [25,27,28,29], and P [30], only genetic information is available. Among the nonclassical lineages, only Z lineage members are expected to bind (probably N-terminally modified) peptides in a way reminiscent of the peptide binding mode of classical MHC-I [28,30].

In the present study, we describe a sixth MHC-I lineage in teleosts. Previously, this lineage had not been noted in teleost fish because of the high level of divergence. We designated the lineage H, after the Greek hexa for six. Teleost H sequences are highly unusual in showing an unprecedented level of deterioration of their ectodomains, while having a large, unique, well-conserved motif in their cytoplasmic tails.

## 2. Materials and Methods

### 2.1. Datamining

A mixture of annotated and un-annotated MHC-I sequences were identified using various blastn and tblastn searches of Ensembl and NCBI databases using evolutionary diverged, as well as species-specific, sequences. Genes, genomic regions, and regional genes were identified using either the Ensembl blast browser: https://www.ensembl.org/index.html [33] (Nile tilapia Orenil1.0; stickleback BROAD S1; tetraodon TETRAODON 8.0; Spotted gar LepOcu 1) or the NCBI genome browser https://www.ncbi.nlm.nih.gov/genome for the remaining species with available genomes (see Supplementary Table S1 for further details). Some open reading frames were predicted using FGENESH [34], aligning genomic and expressed sequences using Splign (https://www.ncbi.nlm.nih.gov/sutils/splign/splign.cgi) and, in some other cases, exons and exon-intron junctions were defined using ORF finder in the Sequence Manipulation suite [35], followed by manual inspection. Deduced amino acid sequences are presented in Supplementary Text S1, while data on genomic sequences and matching expressed sequences are compiled in Supplementary Table S1.

### 2.2. Usage of the Word MHC-I

Our usage of the word “MHC-I” is based on phylogeny [10] and not on location (in the human genome) or function. Therefore, we do not use the distinction between “MHC-I” and “MHC-I-like” as used by some other researchers because we find that to be troublesome when discussing deep MHC evolution across species borders. Among MHC-I molecules, we distinguish between classical and nonclassical based on known or expected presence of classical functions.

### 2.3. Experimental Analysis of Mexican Tetra HAA Transcript Sequences

Total RNA was isolated using TRIzol (Gibco) from the gill and mixed internal organs of a Mexican tetra (*Astyanax mexicanus*) purchased from a pet shop. Animal handling and experiments were in agreement with regulations at Fujita Health University. Total RNA was reverse–transcribed into cDNA using ReverTra Ace (TOYOBO, Osaka, Japan), and the coding sequence of *Asme-HAA* gene was amplified by Ex-Taq HS (Takara Bio, Shiga, Japan) using primers *Asme-HAA 5′UTR.F1* (5′-AAATCATACCTGGGGTCAGCTGTTA-3′) and *Asme-HAA 3′UTR.R1* (5′-GCGAAGCACAACCACATGGTCATGA-3′) designed at 5′ and 3′ untranslated regions, respectively, of the Transcriptome Shotgun Assembly sequence report GFIF01006274. The PCR conditions were: denaturation at 98 °C for 30 s, 40 cycles of denaturation at 98 °C for 10 s, annealing at 54 °C for 30 s, and elongation at 72 °C for 90 s, and final elongation at 72 °C for 3 min. The PCR products were cloned into pGEM-T Easy vector (Promega, Madison, WI, USA) and sequencing reactions were performed with BigDye Terminator v3.1 Sequencing Standard kit (Applied Biosystems, Foster City, CA, USA), and the nucleotide sequences were determined using 3130xl Genetic Analyzer (Applied Biosystems). For both the gill and the mixed internal organ samples, multiple clones were determined to exclude PCR and sequencing artefacts, and for both samples, two different sequences were amplified that only show a single silent nucleotide exchange in the Asme-HAA coding sequence, Asme-HAA*01 and Asme-HAA*02 (Supplementary Text S2), which were deposited at GenBank as accessions LC494124 and LC494125.

### 2.4. Phylogenetic Analysis

Alignments of deduced MHC-I amino acid sequences were made by hand based on considerations on sequence similarity, relatedness of the sequences, and structure as described previously [10]. The transmembrane domain of HLA-A2 was predicted by TMPRED software, https://embnet.vital-it.ch/software/TMPRED_form.html [36]. A phylogenetic tree using the best conserved domain, namely the α2 domain, was inferred using the Neighbor-Joining method [37] with bootstrap testing according to Felsenstein [38]. During the bootstrapping process, 132 failed, so bootstrap values are based on only 868 replicates. Thus, some pairwise distances could not be estimated, such as between sequence Icfu-HAA and Taru-TR6. The evolutionary distances were computed using the p-distance method [39]. The lineage clustering was supported by another phylogenetic tree constructed using Maximum Likelihood method based on the optimal JTT matrix-based model [40] (Supplementary Figure S1). Evolutionary analyses were conducted in MEGA7 [41].

### 2.5. Synonymous Versus Non-Synonymous Nucleotide Substitution Rates

The teleost HAA sequence fragments encoding the unique cytoplasmic tail motif were analyzed by the Synonymous Non-synonymous Analysis Program, SNAP v2.1.1, https://www.hiv.lanl.gov/content/sequence/SNAP/SNAP.html [42]. A similar analysis was performed for the available parts of the full-length coding sequences of characiform *HAA*.

### 2.6. Expression Analyses

Transcriptional values (Reads Per Kilobase per Million mapped reads or RPKM) were calculated using CLC Genomic Workbench 6.0.5 (https://www.qiagenbioinformatics.com/products/clc-genomics-workbench). Reads were mapped with high stringency, i.e., greater than 95% identity over more than 90% of the total length of the query read. Only open reading frame query sequence was used for each gene. Transcriptomes used in this study were: Atlantic salmon (*Salmo salar*) gills (SRR1422858), head kidney (SRR1422860), gut (SRR1422859), ovary (SRR1422871), testis (SRR1422872), spleen (SRR1422870), heart (SRR1422862), brain (SRR1422856), nose (SRR1422867), liver (SRR1422865), skin (SRR1422869), eye (SRR1422857) [43]; Northern pike (*Esox Lucius*) gills (SRR1533653), kidney (SRR1533657), intestine (SRR1533659), ovary (SRR1533651), testis (SRR1533661) [44]; Zebrafish (*Danio rerio*) gills (SRR1524239), kidney (SRR1524243), intestine (SRR1524245), ovary (SRR1524248), testis (SRR1524249); Spotted gar (*Lepososteus oculatus*) gills (SRR1524251), kidney (SRR1524255), intestine (SRR1524257), ovary (SRR1524259), and testis (SRR1524260).

## 3. Results

### 3.1. Identification of H Lineage Sequences in Holostei and Teleostei

**Figure 1:**
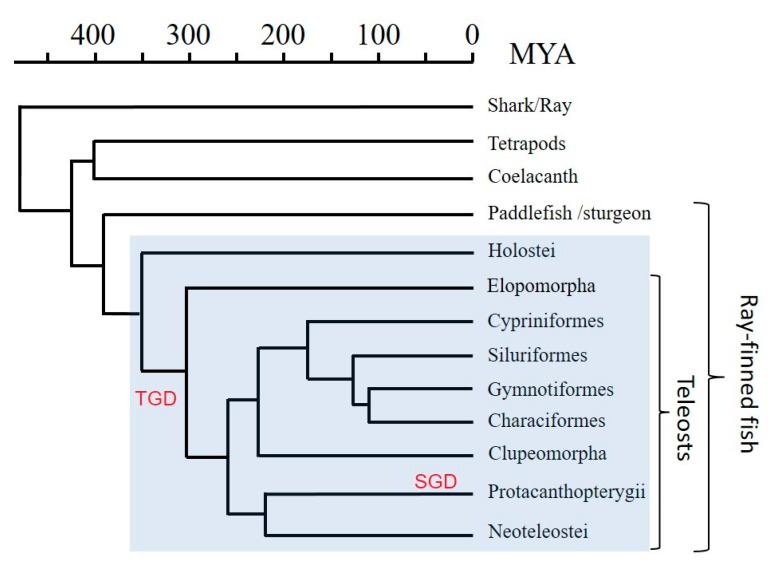
Phylogeny of the fish clades investigated in this study according to Near et al. [46]. A timescale is depicted in millions of years ago (MYA) above the figure. As for the investigated fish species: Holostei include bowfin and spotted gar; Elopomorpha include European eel and American eel; Cypriniformes include common carp, zebrafish, and horned golden-line barbel; Siluriformes include channel catfish, blue catfish, southern catfish, and amur catfish; Gymnotiformes include glass knifefish; Characiformes include Mexican tetra, tambaqui, and pacu; Clupeomorpha include Atlantic herring, Hilsa ilisa, sardine, and allis shad; Protacanthopterygii include Atlantic salmon, rainbow trout, coho salmon, and Northern pike; and Neoteleostei include Nile tilapia, stickleback, tetraodon, yellow croaker, red-lip croaker, guppy, medaka, and turquoise killifish. Clades possessing HAA gene sequences are boxed in blue. The teleost specific third whole genome duplication (TGD) and the salmonid specific fourth whole genome duplication (SGD) are shown.

H lineage sequences were found in Teleostei and Holostei by blast similarity searches using a spotted gar (*Lepisosteus oculatus*) sequence that we previously reported as *LO1* (*Lepisosteus oculatus* sequence 1) [30] and which we now recognize as a member of the previously unknown H lineage and therefore renamed *Leoc-HAA* (nomenclature as suggested in [45]). The sequences presented in the current study were found in public databases (Supplementary Text S2) and, in the case of *Asme-HAA* of Mexican tetra (*Astyanax mexicanus*), also confirmed by experiments (Supplementary Text S2). Phylogeny of the fish clades to which the investigated species belong is shown in Figure 1. Finding of H lineage members in both Holostei and Teleostei implies that the lineage is more than 300 million years old ([46]; Figure 1). We were unable to find H lineage members in other clades of species.

### 3.2. Genomic Positions of Detected H Lineage Genes Reveal Orthology

**Figure 2:**
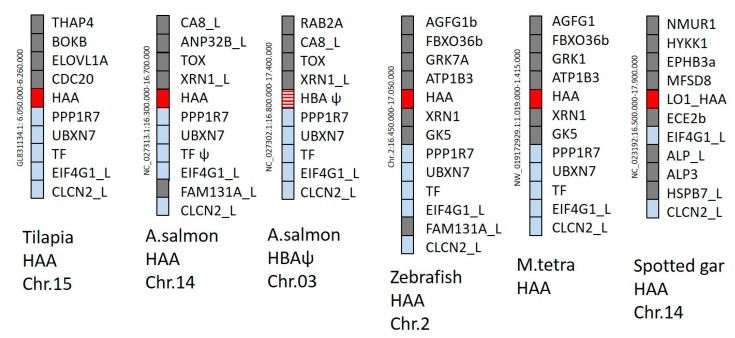
Genomic locations of H lineage loci in representative teleost fishes and spotted gar. H lineage genes and their flanking genes in Tilapia (*Oreochromis niloticus*), Atlantic salmon (*Salmo salar*), Zebrafish (*Danio rerio*), Mexican tetra (*Astyanax mexicanus*), and Spotted gar (*Lepisosteus oculatus*) are shown as boxes. In Atlantic salmon, the region is duplicated and found on two different chromosomes. Red boxes represent intact H lineage genes, striped red represents a probable H lineage pseudogene, and light blue shading is used for non-MHC genes present in three or more species, while grey shading represents non-MHC genes showing poorer conservation in this region. The database sequence position of the depicted genomic region is shown in small font on the left side of each region, and if known the chromosome number is shown at the bottom of the figure. The ψ symbol indicates a probable pseudogene.

Genomic positions of representative H lineage genes are shown in Figure 2 and reveal orthology of the *HAA* genes in teleost fish and spotted gar. In the present study, in cases where the genomic location is not known, the first H lineage genes detected for a species are also named *HAA* (Supplementary Text S1, Table S1). Salmonid fishes experienced a whole genome duplication early in their evolution (e.g., [43,47]), which explains the gene duplication and the presence of *HAA* and a similar *HBA* gene in similar genetic surroundings (Figure 2 and Supplementary Table S1). In Atlantic salmon and rainbow trout (Figure 2 and Supplementary Text S1), but potentially not in coho salmon (*Oncorhynchus kisutch*; Supplementary Text S1), the *HBA* became a probable pseudogene. In common carp (*Cyprinus carpio*) two H lineage loci, *HAA* and *HBA*, are situated closely together (Table S1), indicating their origin by tandem duplication.

The H lineage loci appear not to be linked with the classical *Mhc* region (e.g., compare the H loci positions in Figure 2 with the positions of the classical MHC-I loci in reference [30]), which is quite common among nonclassical MHC-I (e.g., [10,30]).

### 3.3. Intron-Exon Organization of H Lineage Genes and Losses of Ectodomain Exons

**Figure 3:**
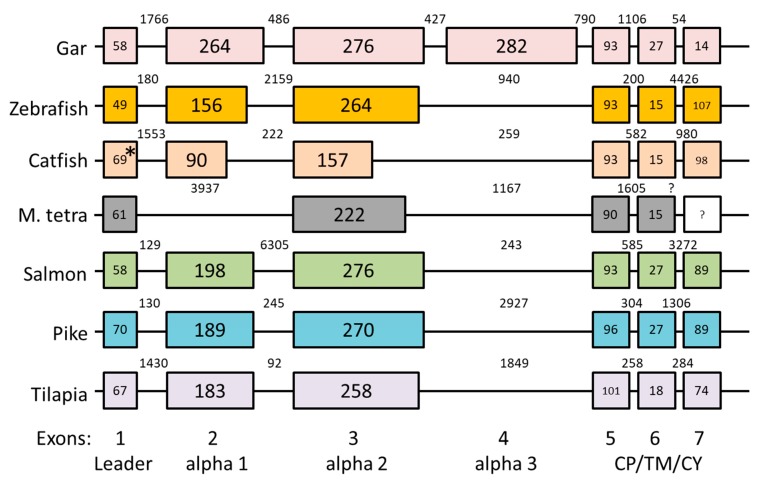
Exon-intron organization of selected *HAA* genes. Exons are shown as differently colored boxes for H lineage genes in Spotted gar (*Lepisosteus oculatus*), Zebrafish (*Danio rerio*), Channel catfish (*Ictalurus punctatus*), Mexican tetra (*Astyanax mexicanus*), Atlantic salmon (*Salmo salar*), Northern pike (*Esox Lucius*), and Tilapia (*Oreochromis niloticus*). The last exon of Mexican tetra found in different cDNA sequences was not present in the currently assembled GenBank genome sequence (Supplementary Text S1 and Text S2) and thus is presented as an uncolored box with question marks as to exon and intron sizes. Because of small differences between genomic and cDNA sequence reports, it was impossible to correctly align the first exon-intron boundary of the catfish *HAA* gene sequence, which is highlighted by an asterisk. Numbers within blocks indicate nucleotide lengths of the coding parts of exons, and numbers between blocks indicate intron lengths. Numbers at the bottom of the figure refer to MHC-I exon consensus numbers, with a description of their encoded domains.

**Figure 4:**
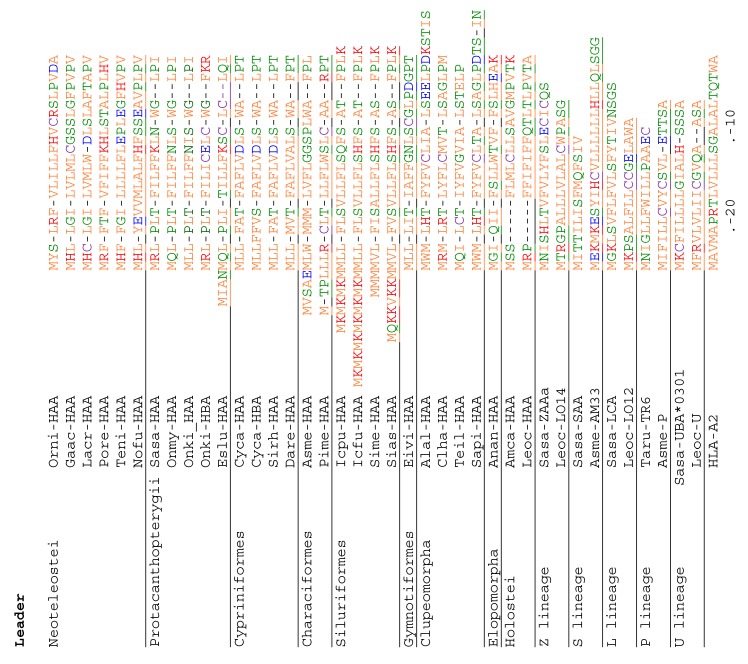

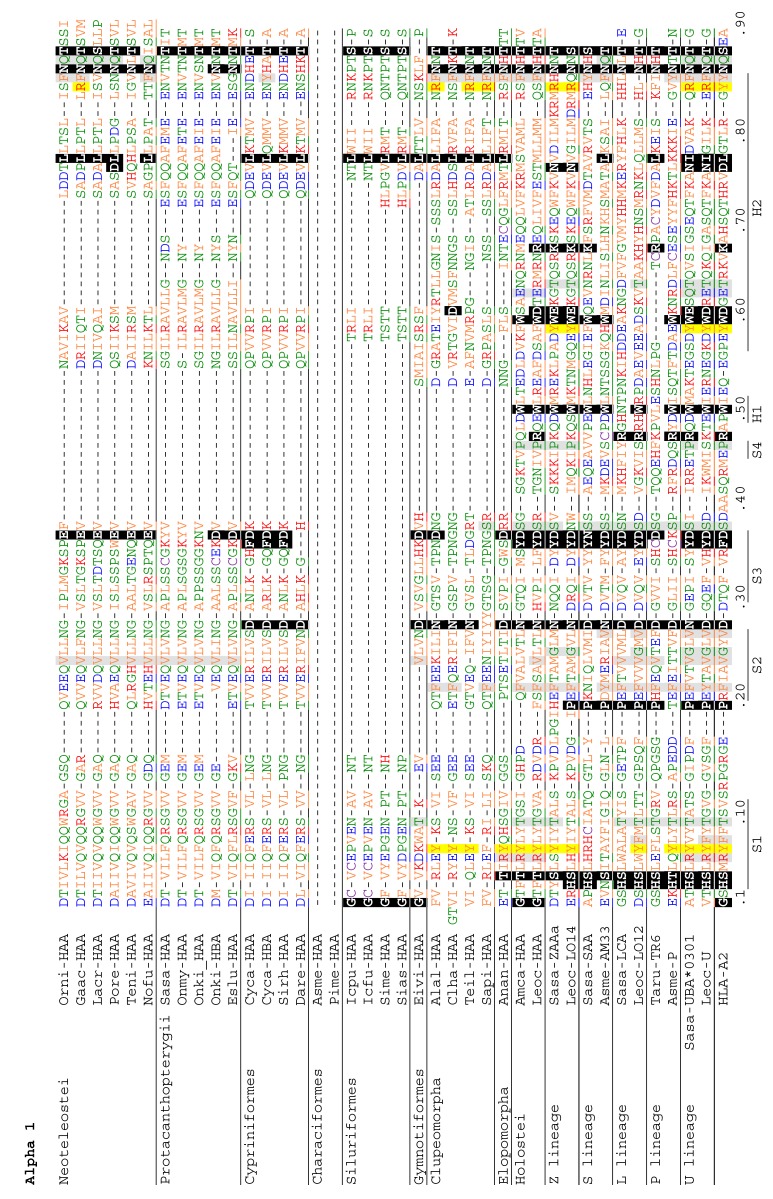

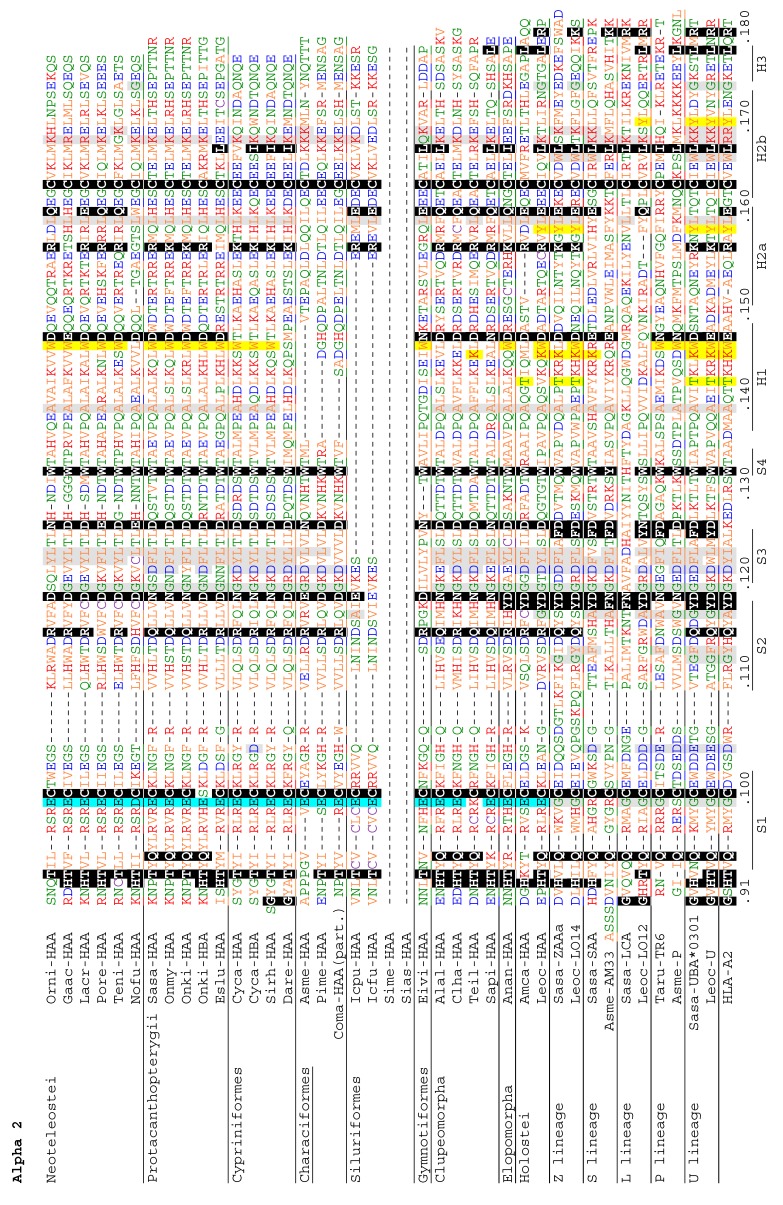

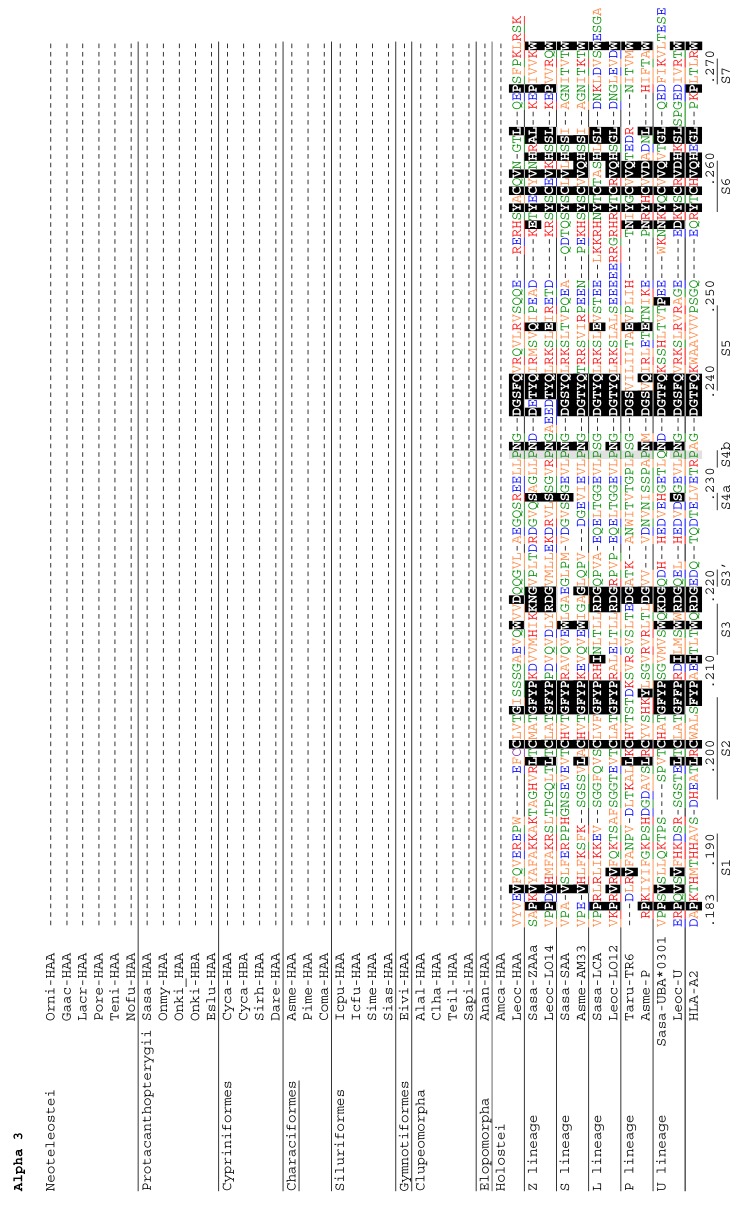

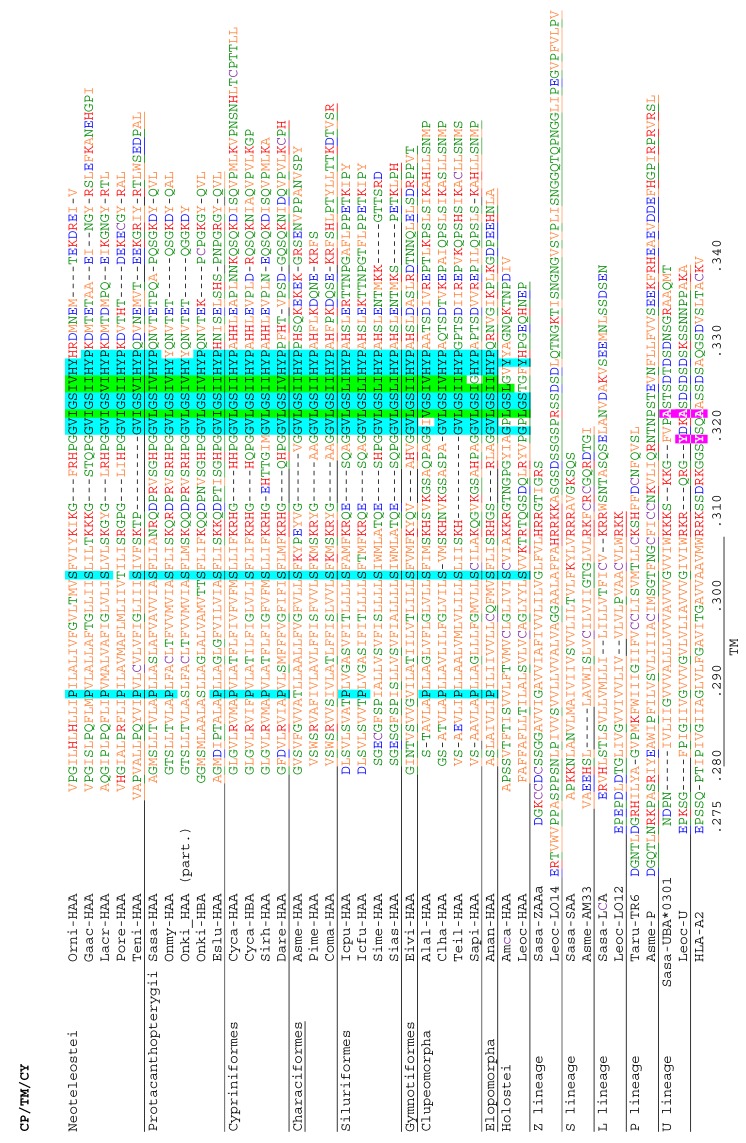
Alignment of deduced H lineage amino acid sequences with other representative MHC-I sequences. Color shading of residues highlights conserved features and is explained in the main text. The sequences are divided into leader, α1, α2, α3, and CP/TM/CY regions, the borders of the first four domains being defined by (expected) exon borders. Numbers under the alignment are based on residue positions in mature HLA-A2 protein, and β-strand (S) and helix (H) structural indications are based on the pHLA-A2 structure in the PDB database accession 3PWN. Non-shaded cysteines are in purple font. Font colors of non-shaded residues are based on reference [48], with basic residues in red, acidic residues in blue, and green residues being more hydrophilic than the orange ones. The (part.) indications refer to incomplete sequence information. H sequence references are as follows: Orni (*Oreochromis niloticus*, Nile tilapia) -HAA (TSA: GBAZ01123113); Gaac (*Gasterosteus aculeatus*, Stickleback) -HAA (DW655318); Lacr (*Larimichthys crocea*, Yellow croaker) –HAA (XP_010741942.1); Pore (*Poecilia reticulata*, Guppy) -HAA (XP_008432358.1 and TSA: GFHH01045885); Teni (*Tetraodon nigroviridis*, Tetraodon) -HAA (CAG07665.1); Nofu (*Nothobranchius furzeri*, Turquoise killifish) -HAA (JZ213307); Sasa (*Salmo salar*, Atlantic salmon) -HAA (XP_013995094.1); Onmy (*Oncorhynchus mykiss*, Rainbow trout) -HAA (XP_021468778.1); Onki (*Oncorhynchus kisutch*, Coho salmon) -HAA (TSA: GDQG01022519.1) and -HBA (TSA: GDQG01022514.1); Eslu (*Esox Lucius*, Northern pike) -HAA (XP_010881869); Cyca (Cyprinus carpio, Common carp) -HAA (KTG41314) and -HBA (KTG33590); Sirh (*Sinocyclocheilus rhinocerous*, Horned golden-line barbel) -HAA (Genomic sequence NW_015649561.1:87.947-91.348); Dare (*Danio rerio*, Zebrafish) -HAA (XM_003197841); Asme (*Astyanax mexicanus*, Mexican tetra) -HAA (LC494124); Pime (*Piaractus mesopotamicus*, Pacu) -HAA (Bioproject PRJEB6656:); Coma (*Colossoma macropomum*, tambaqui) -HAA(TSA: GGHL01056846 and Bioproject PRJNA292457); Icpu (*Ictalurus punctatus*, Channel catfish) -HAA (TSA: JT437950); Icfu (*Ictalurus furcatus*, Blue catfish) -HAA (Bioproject PRJNA195453); Sime (*Silurus meridionalis*, Southern catfish) -HAA (Bioproject PRJNA427243); Sias (*Silurus asotus*, Amur catfish) -HAA (TSA: GHGF01004423); Eivi (*Eigenmannia virescens*, Glass knifefish) -HAA (TSA: GGGZ01064726); Alal (*Alosa*, Allis shad) -HAA (TSA: GETY01043622); Clha (*Clupea harengus*, Atlantic herring) -HAA (Genomic sequence NW_012220971.1: 1.071.225-1.073.475); Teil (*Tenualosa ilisha*, Hilsa ilisa) -HAA (QYSC01123722.1: 356.174-357.933); Sapi (*Sardina pilchardus*, Sardine) -HAA (TSA: GGSC01229082); Amca (*Amia calva*, Bowfin) -HAA (TSA: GEUG01019669); Leoc (*Lepisosteus oculatus*, Spotted gar) -HAA (XP_015216910). The references of the other sequences are Sasa (*Salmo salar*, Atlantic salmon) -SAA (ACY30362.1), -LCA (XP_013983104.1), -ZAAa (ACX35596.1), -UBA (*0301, XP_014032819); Leoc (*Lepisosteus oculatus*, Spotted gar) -Z (LO14, TSA: GFIM01040660), -L (LO12, JH591577:52,184-56,541), -U (TSA: GFIM01032149); Asme (*Astyanax mexicanus*, Mexican tetra) -S (AM33, ENSAMXG00000017444), -P (TSA: GFIF01000014); Taru (*Takifugu rubripes*, Fugu) -P (Scaffold_497:44,782-49,340); and Human HLA-A2 (AAA76608.2). See Supplementary Table S1 and Text S1 for more details.

Comparison of available genomic and cDNA information (Supplementary Table S1) allowed analysis of intron-exon organization. Intron-exon organizations of representative H lineage genes are shown in Figure 3, and sequences encoded by the α1, α2, and α3 exons are separately aligned in Figure 4. Spotted gar *Leoc-HAA* encodes all domains of a consensus MHC-I molecule, including α1, α2, α3, and CP/TM/CY domains, and the intron-exon organization is as commonly found among MHC-I genes (Figure 3). In contrast, cDNA analysis indicates that teleost fish H lineage genes do not possess α3 domain exon sequences (Figure 4 and Supplementary Text S1) and analysis of the genomic region sequences confirms this absence (Figure 3). Furthermore, in neither cDNA (Figure 4; Supplementary Text S2) nor genomic DNA (Figure 3) could an α1 domain exon sequence be found for Mexican tetra *Asme-HAA*, and this α1 absence was confirmed at the cDNA level for another characiform fish, pacu (*Piaractus mesopotamicus*) (Figure 4; Supplementary Text S1). Characiformes are related with Siluriformes and Gymnotiformes (Figure 1; [46]), and in Siluriformes, the deterioration of a consensus type MHC-I ectodomain is also pronounced. For example, in the two investigated fish of the genus *Silurus*, Southern catfish (*Silurus meridionalis*) and Amur catfish (*Silurus asotus*), the α2 exon appears to be entirely lacking according to investigated cDNA sequences (Figure 4; Supplementary Text S1); however, genomic information for these *Silurus* H genes is absent and, theoretically, there might be additional transcripts from the same genes that do include an α2 exon. Yet, for the related siluriform species channel catfish (*Ictalurus punctatus*), both cDNA and genomic sequence information is available (Supplementary Table S1), providing solid evidence that large parts of both α1 and α2 exon consensus were lost (Figure 3 and Figure 4), and this probably relates to the same lack of importance of the ectodomains as reflected in the complete loss of α1 exon sequences in characiform H and of α2 exon sequences in *Silurus* H. Lack of conservation pressure for maintaining H lineage ectodomains may also explain the short α1 and α2 sequences of *Eivi-HAA* of glass knifefish (*Eigenmannia virescens*) belonging to Gymnotiformes (Figure 4), although the fragment losses are less extreme compared to those in Characiformes and Siluriformes. Teleost fishes other than Characiformes/Siluriformes/Gymnotiformes (C/S/G) possess H lineage sequences with an α2 length that is quite similar to MHC-I consensus, but their α1 sequence length is considerably shorter than the consensus, although not as short as in H sequences in C/S/G fish (Figure 3 and Figure 4).

### 3.4. Deduced Amino Acid Sequences of Teleost H Lineage Molecules Reveal Deterioration of Ectodomains and the Possession of an Unusual Cytoplasmic Tail Motif

In Figure 4, the deduced amino acid sequences of H lineage sequences are compared with representative sequences of other MHC-I lineages found in teleost fish, and with the human classical MHC-I molecule HLA-A2. Black shading highlights residues or sets of similar residues that probably were also present before the evolutionary separation of the MHC classes I and II [10,49], gray shading highlights residues or sets of similar residues that are common in and rather specific for classical MHC-I ([10] and our ongoing investigations), and yellow shading highlights conserved residues which in classical MHC-I are involved in binding the peptide termini [3,5]. The depicted Atlantic salmon Sasa-UBA*0301 sequence is an allele of the polymorphic classical *UBA* locus [30,50]. From its length and shading pattern in Figure 4, it is readily concluded that spotted gar Leoc-HAA shows an overall similarity to classical MHC-I and may build a similar structure, whereas it lost four of the classical MHC-I residues used for binding of peptide termini and possibly does not possess a groove for peptide binding. Holostei are classified into the orders Lepisosteiformes and Amiiformes, represented by spotted gar and bowfin (*Amia calva),* respectively. Although an α3 domain is present in spotted gar Leoc-HAA, in bowfin Amca-HAA and teleost H lineage sequences, the α3 domain is absent (Figure 2), which suggests that the α3 domain was independently lost in the bowfin and teleost fish ancestors. However, the absence of an α3 domain in bowfin Amca-HAA would need confirmation at the genomic level because the single available cDNA sequence might represent only one of multiple splicoform variants.

**Figure 5:**
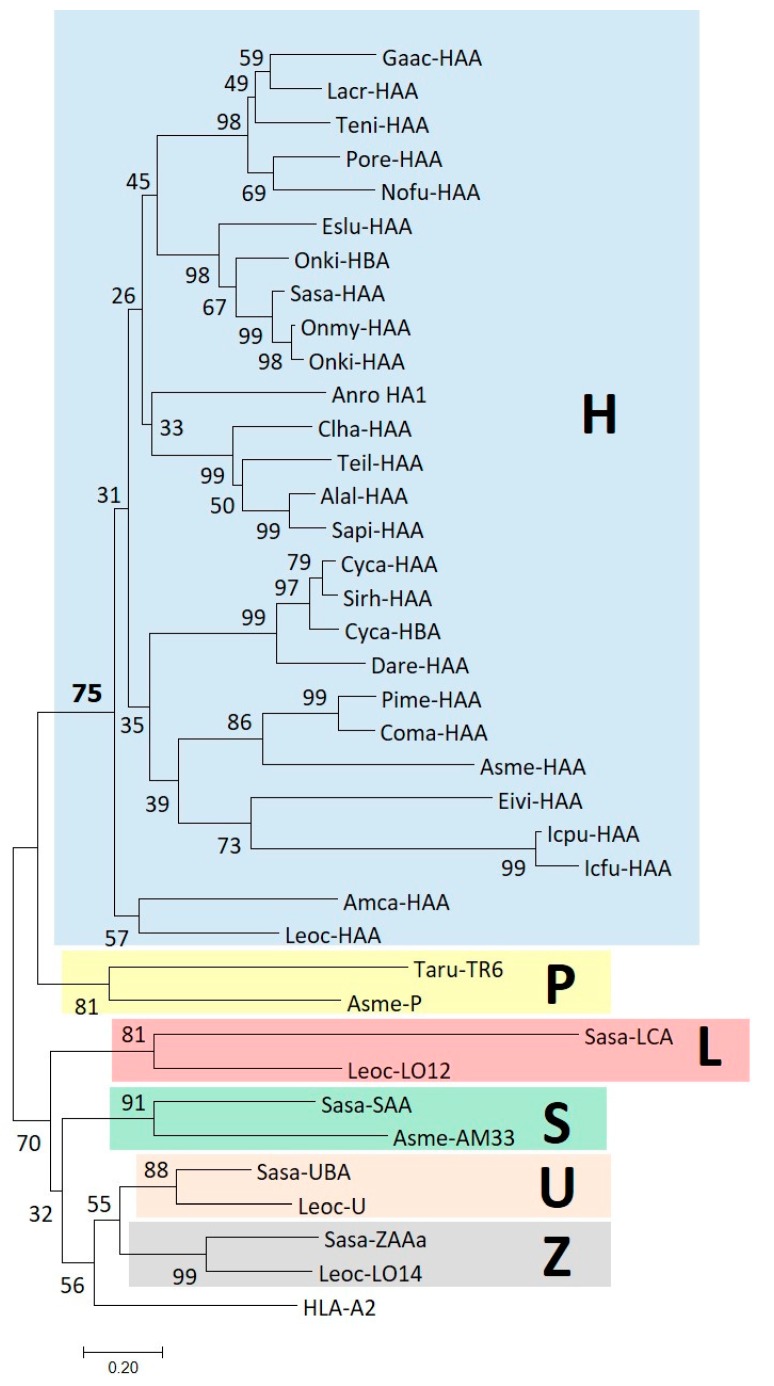
Phylogenetic tree based on α2 domain amino acid sequences of representative H lineage and other MHC-I molecules as aligned in Figure 4. Clusters with the six different MHC class I lineages found in teleosts are shown with colored boxes. The evolutionary history was inferred using the Neighbor-Joining method [37]. The optimal tree with the sum of branch length = 15.21024459 is shown. The percentage of replicate trees in which the associated taxa clustered together in the bootstrap test (1000 replicates) are shown next to the branches [38]. The tree is drawn to scale, with branch lengths in the same units as those of the evolutionary distances used to infer the phylogenetic tree. The evolutionary distances were computed using the Poisson correction method [39] and are in the units of the number of amino acid substitutions per site. The analysis involved 38 amino acid sequences. All ambiguous positions were removed for each sequence pair. There were a total of 104 positions in the final dataset. Evolutionary analyses were conducted in MEGA7 [41]. Sequence references can be found in legend to Figure 4 and in Supplementary Text S1.

Because of the large degree of diversification between the sequences, some regions of the Figure 4 alignment are only tentative. Except for the apparent and differential losses of ectodomain parts, it is difficult to find common features that collectively distinguish the H lineage ectodomains from other MHC-I lineages. The only readily distinguishable specific feature may be the acidic residue at position 100, shaded blue in Figure 4. Nonetheless, phylogenetic tree analysis shows that the H lineage α2 domain sequences do share overall specific similarity as the H sequences form a single cluster (Figure 5; we refrained from such analysis for the α1 domain because of uncertainty about the correct alignment).

Among classical MHC-I cytoplasmic tails, the YXXA motif (X denotes any possible residue) is rather well conserved (shaded magenta in Figure 4), although in teleost fish the tyrosine is commonly replaced by phenylalanine (Figure 4; [51]). The YXXA motif in classical MHC-I plays a role in endocytic trafficking of surface MHC-I molecules and the loading with exogenous antigens [52,53]. These residues are not present in the H lineage sequences.

However, in sharp contrast to the diversification of their ectodomains, the teleost H lineage sequences show remarkable conservation of a large motif in the cytoplasmic tail GV(I/L)GS(I/L/V)(I/V)HYP. In Figure 4, the single residues within this motif in teleost H are shaded blue and the variable residues are shaded green. We are not aware of a similar cytoplasmic tail motif in other proteins, but the conserved tyrosine suggests involvement in intracellular pathways by means of phosphorylation. The conservation of hydrophobic residues at some positions within the cytoplasmic tail motif suggests interaction with another as yet unidentified protein. Equally, the conservation of a hydrophilic serine at position 304 in the teleost H lineage transmembrane domain sequences, and maybe also the partial conservation of a proline at position 290, suggest interaction of the TM domain with some other molecule. Examples of molecules believed to use a conserved serine/threonine or proline within the transmembrane domain for intermolecular protein binding are CD74 [54,55] and Igα [56], respectively. The identity of the molecules putatively interacting with the HAA TM/CY regions can only be guessed. In H molecules of Holostei, only parts of the teleost H lineage GV(I/L)GS(I/L/V)(I/V)HYP cytoplasmic tail motif are found, but, importantly, the tyrosine within the motif is conserved, as is the unusual serine within the TM domain. Thus, unique features of the H lineage transmembrane and cytoplasmic tail domains have been conserved over 300 million years, underlining their probable importance.

### 3.5. Nucleotide Sequences Encoding the Teleost H Lineage Cytoplasmic Tail Motif Indicate Purifying Selection

A possible explanation for the deterioration of the H lineage ectodomains in teleosts could be that the proteins lost their function and that the teleost H genes and gene transcripts are only nonfunctional remnants of an evolutionary past, or, alternatively, that teleost H genes only have a function at the transcript level. Because a lack of protein function is reflected in the rates of synonymous versus non-synonymous (ds versus dn) substitutions that accumulate in a gene, the nucleotide sequences encoding the cytoplasmic tail GV(I/L)GS(I/L/V)(I/V)HYP motif were compared between all teleost H lineage molecules shown in Figure 4 and between the H lineage molecules of only Characiformes/Siluriformes/Gymnotiformes (C/S/G) (Supplementary Text S3A–C). The results in Supplementary Text S3 show that the ds/dn ratio among H lineage molecules of all compared teleost aligned is 14.60 and that, among the H lineage molecules of the C/S/G teleost subgroup, it is 20.03. These numbers indicate purifying selection at the amino acid level and suggest that H lineage proteins are functional in teleosts, including in C/S/G fish.

The characiform *HAA* sequences *Asme-HAA*, *Pime-HAA*, and *Coma-HAA* are sufficiently similar and dissimilar for allowing reliable alignment and meaningful ds/dn analysis for the available part of their full-length coding sequences, and the calculated ds/dn ratios were 2.00 for *Asme*/*Pime*, 2.35 for *Asme*/*Coma*, and 1.71 for *Pime*/*Coma* (Supplementary Text S3D). This is additional evidence for purifying selection at the amino acid level in characiform H lineage sequences, even after losses of both the α1 and α3 domains.

### 3.6. Expression Pattern

The tissue distribution of H lineage transcripts was determined by analysis of transcriptome data. Initially, we investigated a panel with a wide variety of Atlantic salmon tissue transcriptomes available in GenBank [43]. This analysis showed highest expression in Atlantic salmon ovary with low to medium expression levels in other tissues, disregarding liver and skin where the expression levels were insignificant (Table 1). The expression levels are comparable to those of other non-classical Atlantic salmon MHC-I genes, such as *UDA*, *LDA*, and *ZBAa*, and are generally much lower than found for the classical *UBA* gene (Table 1, data from [30]). We then analyzed the expression of *HAA* genes in transcriptome data publicly available for several ray-finned fish species and found an overall low to medium expression level (Table 1). The high expression level found in Atlantic salmon ovary was neither seen in ovary samples from the other species shown in Table 1 nor in rainbow trout ovary (GenBank ERR324375; rainbow trout data not shown). However, in general, it is difficult to compare transcription values between animals and samples as the biological age and status of the animals, providing the transcriptomes are not well defined. From Table 1, it can be concluded that H lineage genes are transcribed in most tissues in species ranging from spotted gar to Atlantic salmon, but the detected expression pattern is not helpful for predicting a specific function.

## 4. Discussion

In the present study, a sixth lineage of teleost MHC-I sequences is presented and designated H. H lineage sequences are also found in Holostei, and spotted gar HAA at least looks structurally similar to classical sequences. Spotted gar HAA has all domains found in classical MHC-I, and those domains are of similar length and have many of the MHC-I and MHC-I/II characteristic residues (gray and black shading in Figure 4), although several of the classical residues for binding of peptide termini (yellow shading in Figure 4) were lost. In contrast to the spotted gar HAA sequence, the teleost H lineage sequences show an unprecedented deterioration of the canonical MHC-I ectodomain structure, with a loss of the α3 domain and a seemingly random loss of stretches and residues within the α1 and α2 domains, or even of the entire α1 or α2 domains. Although firm conclusions cannot be drawn, the impression from the sequence comparisons is that, in teleost H lineage molecules, the ectodomain lost most of its function. In contrast, unique residues in the transmembrane domain and cytoplasmic tail which are found in Holostei H molecules are also conserved in teleosts and suggest binding of a yet unknown partner molecule and intracellular signaling through tyrosine phosphorylation. Especially among teleost H molecules, the conservation of a large cytoplasmic motif including the mentioned tyrosine is impressive. We are not aware of any previous descriptions of a group of MHC molecules with such deteriorated ectodomains or with such an impressive cytoplasmic tail motif. Regarding the often posed question of how the unique MHC peptide-binding domain structure emerged in evolution the teleost H sequences are quite interesting, as they seem to provide evidence that also partial MHC structures can be stable. Therefore, future studies should not only investigate the function but also the structures of the H lineage molecules.

## Figures and Tables

**Table 1:** *HAA* transcription in various tissues of several teleost fish species. Transcriptional values are given as RPKM, i.e., Reads Per Kilobase per Million mapped reads. See Materials and Methods section for dataset references. * Tissue denoted kidney is defined as head kidney in Atlantic salmon but as kidney in the other species. ** Intestine/gut is defined as gut in Atlantic salmon but as intestine in the other species. Atlantic salmon RPKM values in grey colored cells for the classical *UBA* locus and the nonclassical *UDA*, *LDA*, and *ZBAa* genes originate from our previous study [30].

Tissue\Gene	Sasa-HAA	Eslu-HAA	Dare-HAA	Leoc-HAA	Sasa-UBA	Sasa-UDA	Sasa-LDA	Sasa-ZBAa
Gills	9.48	1.57	3.50	0.95	253.57	5.65	3.36	43.46
Kidney *	11.49	1.79	4.11	3.55	66.97	4.05	4.17	9.42
Intestine/gut **	16.47	1.90	4.41	11.09	361.72	4.27	1.86	18.57
Ovary	56.84	2.66	8.65	1.35	1.25	4.29	6.08	0.11
Testis	10.49	1.54	3.18	2.07	60.84	6.31	1.14	2.70
Spleen	9.62	n/a	n/a	n/a	260.43	6.63	2.72	19.47
Heart	2.85	n/a	n/a	n/a	16.79	1.35	1.24	8.11
Brain	5.61	n/a	n/a	n/a	17.65	1.75	0.30	3.34
Nose	2.40	n/a	n/a	n/a	60.70	5.44	2.02	8.61
Liver	0.94	n/a	n/a	n/a	12.92	0.78	1.23	4.30
Skin	0.17	n/a	n/a	n/a	4.84	6.31	0.09	0.08
Eye	1.77	n/a	n/a	n/a	11.75	0.79	0.15	2.76
Query Length (bp)	741	741	693	1056	1068	1068	1080	1128

## Supplementary Materials

The following are available online at https://www.mdpi.com/2073-4409/8/9/1056/s1, Text S1: Gene specifics and sequences; Text S2: Mexican tetra amplification; Text S3: Substitution rates; Table S1. Genomes, genomic location and expressed match; and Figure S1. Maximum Likelihood phylogenetic tree.
