# Supplementary material for: Discovery of a Novel MHC Class I Lineage in Teleost Fish which Shows Unprecedented Levels of Ectodomain Deterioration while Possessing an Impressive Cytoplasmic Tail Motif
Source: Cells. 2019 Sep 9;8(9):1056. doi: 10.3390/cells8091056 (PMC6769792; doi:10.3390/cells8091056)
Supplement: Supplementary file 1 [file cells-08-01056-s001.zip › Supplementary Figure S1. ML tree.pdf]

Supplementary Figure S1: Molecular phylogenetic analysis of α2 domain sequences by Maximum Likelihood method

The evolutionary history of the α2 domain sequences of the MHC-I sequences aligned in main text Fig. 4 was inferred by using the Maximum Likelihood method based on the JTT matrix-based model [1]. The tree with the highest log likelihood (-2726.73) is shown (page 2). The percentage of trees in which the associated taxa clustered together is shown next to the branches. Initial tree(s) for the heuristic search were obtained automatically by applying Neighbor-Join and BioNJ algorithms to a matrix of pairwise distances estimated using a JTT model, and then selecting the topology with superior log likelihood value. A discrete Gamma distribution was used to model evolutionary rate differences among sites (5 categories (+G, parameter = 8.4828)). The rate variation model allowed for some sites to be evolutionarily invariable ([+I], 2.16% sites). The tree is drawn to scale, with branch lengths measured in the number of substitutions per site. The analysis involved 38 amino acid sequences. All positions with less than 95% site coverage were eliminated. That is, fewer than 5% alignment gaps, missing data, and ambiguous bases were allowed at any position. There were a total of 46 positions in the final dataset. Evolutionary analyses were conducted in MEGA7 [2].

1. Jones D.T., Taylor W.R., and Thornton J.M. (1992). The rapid generation of mutation data matrices from protein sequences. Computer Applications in the Biosciences 8: 275-282.
2. Kumar S., Stecher G., and Tamura K. (2016). MEGA7: Molecular Evolutionary Genetics Analysis version 7.0 for bigger datasets. Molecular Biology and Evolution 33:1870-1874.

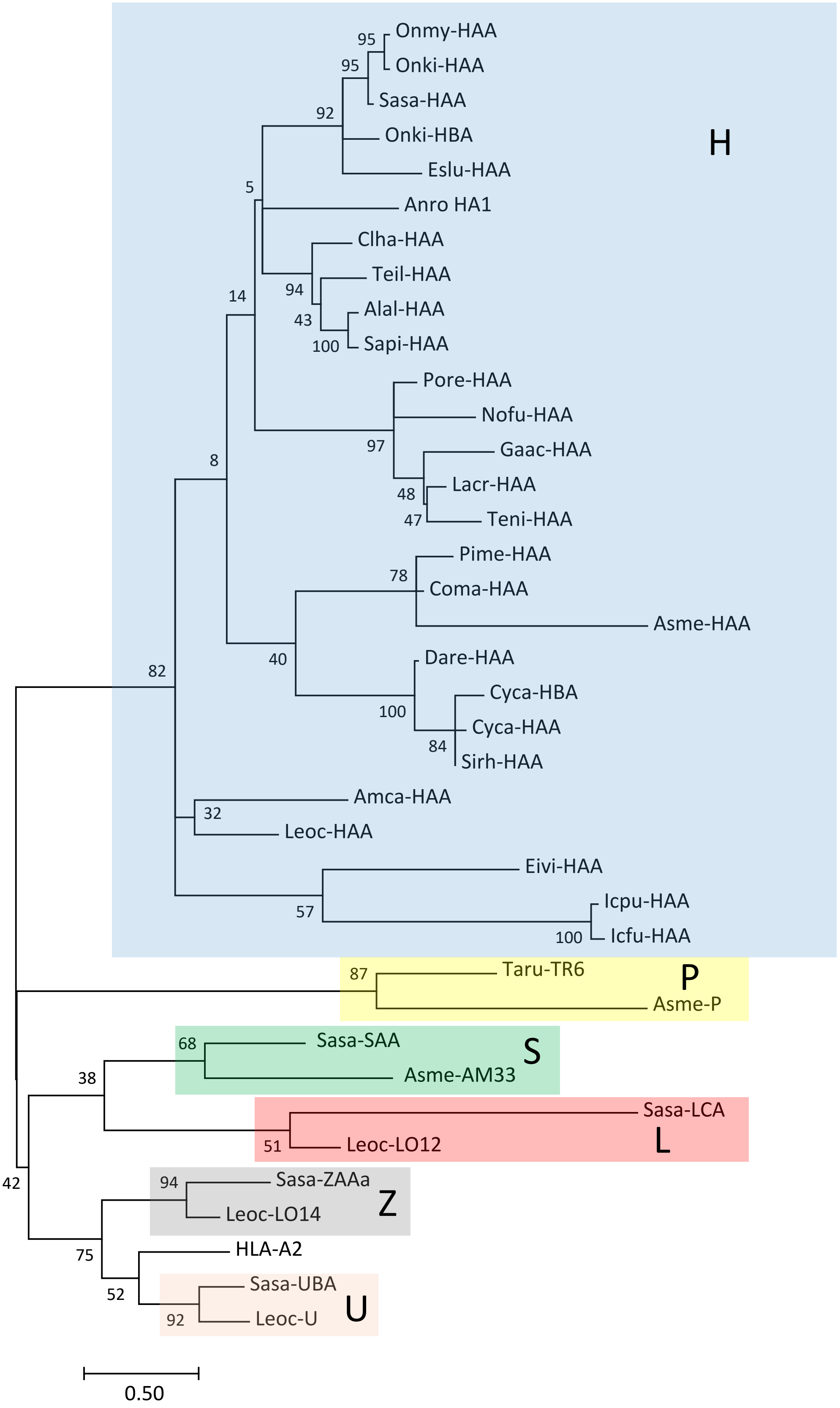
H
Onmy-HAA
Onki-HAA
Sasa-HAA
Onki-HBA
Eslu-HAA
Anro HA1
Clha-HAA
Teil-HAA
Alal-HAA
Sapi-HAA
Pore-HAA
Nofu-HAA
Gaac-HAA
Lacr-HAA
Teni-HAA
Pime-HAA
Coma-HAA
Asme-HAA
Dare-HAA
Cyca-HBA
Cyca-HAA
Sirh-HAA
Amca-HAA
Leoc-HAA
Eivi-HAA
Icpu-HAA
Icfu-HAA
P
Taru-TR6
Asme-P
S
Sasa-SAA
Asme-AM33
L
Sasa-LCA
Leoc-LO12
Z
Sasa-ZAAa
Leoc-LO14
HLA-A2
U
Sasa-UBA
Leoc-U
0.50
