# Supplementary material for: Discovery of a Novel MHC Class I Lineage in Teleost Fish which Shows Unprecedented Levels of Ectodomain Deterioration while Possessing an Impressive Cytoplasmic Tail Motif
Source: Cells. 2019 Sep 9;8(9):1056. doi: 10.3390/cells8091056 (PMC6769792; doi:10.3390/cells8091056)
Supplement: Supplementary file 1 [file cells-08-01056-s001.zip › Supplementary Table S1. Genomes, genomic locations and expressed match.docx]

**Supplementary Table S1. Genomic location and/or expressed match/ Bioproject for H lineage gene sequences in ray-finned fishes**

| H lineage genes in various species | Genomic location | Predicted Gene ID | Expressed support | NCBI/ Ensembl Genome version |
| --- | --- | --- | --- | --- |
| **Neoteleostei** | | | | |
| *Oreochromis niloticus*  Orni-HAA  Nile tilapia | GL831134.1: 6.225.747-6.232.374 | ENSONIG00000022885 (orf error, i.e. no protein predicted) | TSA:GBAZ01123113 | Orenil1.0;GCA_000188235.1 |
| *Gasterosteus aculeatus*  Gaac-HAA  Stickleback | Group III: 1.630.711-1.634.932 | ENSGACG00000013621 | DW655318 | BROAD S1 |
| *Tetraodon nigroviridis*  Teni-HAA  Tetraodon | Chr.15:5.789.561-5.792.571 | CAG07665.1 | CR638748.2 | TETRAODON 8.0 |
| *Larimichthys crocea*  Lacr-HAA  Large yellow croaker | NW_017608489.1:2.035.842-2.039.683 | LOC104929182 | *Larimichthys polyactis* TSA: GETG01010010.1 | GCA_000972845.1 |
| *Poecilia reticulata* Pore-HAA  Guppy | NC_024347.1:18.250.846-18.254.150 (LG17) | XP_008432358.1  (LOC103479603) | TSA:GFHH01045885.1 | GCA_000633615.2 |
| *Nothobranchius furzeri*  Nofu-HAA  Turquoise killifish | 0 | 0 | JZ213307 | 0 |
| **Protacanthopterygii** (Salmonidae/ Esociformes) | | | | |
| *Salmo salar*  Sasa-HAA  Atlantic salmon | NC_027313.1:16.480.799-16.491.490 (Chr.14) | XP_013995094.1 | TSA:GEGX01039681 | GCA_000233375.4 |
| *Salmo salar*  Sasa-HBAψ  Atlantic salmon | NC_027302.1:17.030.685-17.037.352 (Chr.03) | No Gene ID;  Probable pseudogene | No match | GCA_000233375.4 |
| *Oncorhynchus mykiss*  Onmy-HAA Rainbow trout | NC_035084.1: 52.835.011-52.852.788 (Chr.8) | XP_021468778.1 | CU063683.1 | GCA_002163495.1 |
| *Oncorhynchus mykiss*  Onmy-HBAψ  Rainbow trout | NC_035104.1: 26.645.363-26.651.156 (Chr.28) | No gene ID | No match | GCA_002163495.1 |
| *Oncorhynchus kisutch*  Onki-HAA  Coho salmon | NC_034203:15.300.093-15.319.385 (LG30) | XP_020322949.1 | TSA:GDQG01022517.1 | GCA_002021735.1 |
| *Oncorhynchus kisutch*  Onki-HAA ψ  Coho salmon | NC_034203.1: 15.290.463-15.297.915 (LG30) | No Gene ID | No match | GCA_002021735.1 |
| *Oncorhynchus kisutch*  Onki-HBA  Coho salmon | NC_034200:25.424.828-25.439.103 (LG27) | XP_020319115.1 | No match | GCA_002021735.1 |
| *Esox lucius*  Eslu-HAA  Northern pike | NC_025970.2: 290.003-295.584 (LG03) | XP_010881876.1 | No match | GCA_000721915.3 |
| **Cypriniformes** | | | | |
| Danio rerio  Dare-HAA  Zebrafish | Chr.2:16.585.549-16.595.596 | ENSDARG00000092225 | TSA:GFIL01014849.1 | GRCz11;GCA_000002035.4 |
| *Cyprinus carpio* Cyca-HAA  Common carp | Chr.4:5.071.107-5.074.000 | XP_018979243.1 | TSA:GFWU01041615.1 | GCA_001270105.1 |
| *Cyprinus carpio* Cyca-HBA  Common carp | Chr.4:4.975.004-4.977.896 | No Gene ID | TSA:GFWU01041655.1  (Internal stop codon) | GCA_001270105.1 |
| *Sinocyclocheilus rhinocerous*  Sirh-HAA  Horned golden-line barbell | NW_015649561.1:87.947-91.348 | No Gene ID | No match | GCA_001515625.1 |
| *Sinocyclocheilus rhinocerous*  Sirh-HBA  Horned golden-line barbel | NW_015666593.1: 418.897-426.574 | No Gene ID; Internal stop codon, probable pseudogene | No match | GCA_001515625.1 |
| *Sinocyclocheilus rhinocerous*  Sirh-HCA  Horned golden-line barbel | NW_015662004.1:1.803.008-1.803.959 | No Gene ID; Partial gene, probable pseudogene | No match | GCA_001515625.1 |
| **Characiformes** | | | | |
| *Astyanax mexicanus*  Asme-HAA  Mexican tetra | NW_019172929.1: 1.261.266-1.266.759 | No Gene ID | LC494124 and LC494125 | APWO00000000.2 |
| *Colosomma macripomum*  *Coma-HAA*  Tambaqui | 0 | 0 | TSA:GGHL01056846 and Overlapping SRAs of Bioproject PRJNA292457: SRR2167775.53650192, SRR2167784.51538555 | 0 |
| *Piaractus mesopotamicus*  Pime-HAA  Pacu | 0 | 0 | Overlapping SRAs of Bioproject PRJEB6656: ERR556971.59046231.1, ERR556972.27067064.2, ERR556971.59046231.2, ERR556970.15008529.2, ERR556970.29306149.2, ERR556967.56278181.2, ERR556972.29077573.1, ERR556970.20299803.2, ERR556967.53765824.1 | 0 |
| **Siluriformes** | | | | |
| *Ictalurus punctatus*  Icpu-HAA  Channel catfish | NC_030435.1: 21.975.148-21.977.640 | No gene ID | TSA: JT437950 | GCA_001660625.1 |
| *Ictalurus furcatus*  Icfu-HAA  Blue catfish | 0 | 0 | Overlapping SRAs of Bioproject PRJNA195453: SRR799808.12822488.1, SRR799807.4204303.2, SRR799808.18238282.1, SRR799813.16020909.2, SRR799807.4112183.1, SRR799813.4094285.2, SRR799807.6769313.2, SRR799809.15128930.2, SRR799807.13852084.2, SRR799810.1410347.2  SRR799810.5892360.2 | 0 |
| *Silurus meridionalis*  Sime-HAA  Southern catfish | 0 | 0 | Overlapping SRAs of Bioproject PRJNA427243: SRR7224141.21783031.2, SRR7224141.9580927.1, SRR7224141.22203036.1, SRR7224141.4610823.1, SRR7224145.42121942.1 | 0 |
| *Silurus asotus*  Sias-HAA  Amur catfish | 0 | 0 | TSA: GHGF01004423 | 0 |
| **Gymnotiformes** | | | | |
| *Eigenmannia virescens*  Eivi-HAA  Glass knifefish | 0 | 0 | TSA: GGGZ01064726 | 0 |
| **Clupeomorpha** | | | | |
| *Clupea harengus*  Clha-HAA  Atlantic herring | NW_012220971.1: 1.071.227-  1.073.474 | No gene ID | No match | JZKK00000000.1 |
| *Tenualosa ilisha*  Teil-HAA  Hilsa ilisa | QYSC01123722.1: 356.174-357.933 | No gene ID | No match | GCA_003651195.1 |
| *Sardina pilchardus*  Sapi-HAA  Sardine | 0 | 0 | TSA: GGSC01229082 | 0 |
| *Alosa alosa*  Alal-HAA  Allis shad | 0 | 0 | TSA: GETY01043622 | 0 |
| **Elopomorpha** | | | | |
| *Anguilla anguilla*Anan-HAA European eel | 0 | 0 | TSA: GFIC01029617.1 | 0 |
| *Anguilla rostrata*Anro-HAAAmerican eel | LTYT01001052.1:54.564-57.749 | No Gene ID | No match | LTYT00000000.1 |
| Holostei | | | | |
| *Lepisosteus oculatus*  Leoc-HAA (L01)  Spotted gar | NC_023192.1: 17.405.421-17.409.812  (LG14) | XP_015216910 | TSA:GFIM01030402.1 | LepOcu1;GCA_000242695.1 |
| *Amia calva*  Amca-HAA  Bowfin | 0 | 0 | TSA:GEUG01019669 | 0 |
