# Supplementary material for: Discovery of a Novel MHC Class I Lineage in Teleost Fish which Shows Unprecedented Levels of Ectodomain Deterioration while Possessing an Impressive Cytoplasmic Tail Motif
Source: Cells. 2019 Sep 9;8(9):1056. doi: 10.3390/cells8091056 (PMC6769792; doi:10.3390/cells8091056)
Supplement: Supplementary file 1 [file cells-08-01056-s001.zip › Supplementary Text S1. Deduced amino acid sequences.docx]

**Supplementary Text S1: Deduced MHC class I amino acid sequences**

**Various deduced H lineage amino acid sequences and their source information in the NCBI or Ensembl databases:**

**Neoteleostei:**

>Orni-HAA *Oreochromis niloticus* (Nile tilapia). TSA: GBAZ01123113

MYSLRFVLILLFHVCRSLPVDADTIVLKIQQWRGAGSQQVEEQVLLNGIPLMGKSPEFNA

VIKAVLDDTLLTSLISFNQTSSISNQTILRSRECTWEGSKLRWADRVFADSQLYLTLNHN

DIWTAHVQEAVAIKVVWDQEVQQTRAERLDLQEGCVKLMKHLNPSEKQSVPGILHLHLLI

PILALIVFGVLTMVSFVIYKIKGFRHPGGVIGSIVHYHRDMNEMTEKDREIV

>Orni-HAA *Oreochromis niloticus* (Nile tilapia). TSA: GBAY01103141.1

MYSLRFVLILLFHVCRSLPVDADTIVLKIQQWRGAGSQQVEEQVLLNGIPLMGKSPEFNA

VIKAVLDDTLLTSLISFNQTSSISNQTILRSRECTWEGSKLRWADRVFADSQLYLTLNHN

DIWTAHVQEAVAIKVVWDQEVQQTRAERLDLQEGCVKLMKHLNPSEKQSVPGILHLHLLI

PILALIVFGVLTMVSFVIYKIKGFRHPGGVIGSIVHYHRDMNEMTEKDREIV

>Gaac-HAA *Gasterosteus aculeatus* (stickleback). DW655318

MHLLGILVLMLCGSSLGFPVPVDTILVQVQQRGVVGARQVVEQVLFNGVSLTGKSPEVDR

IIQTISADPLLPTLLRFNQTSVMRDHTVFRSRECIVEGSLLHWADRVFYDGEVYLTLDHG

GGWTPRVPEALAFKVVWEQQEQRTKRETSHLHEGCIKLMRELMLSQEQSVPGISLPQFLM

PVLALLAFTGLLIISLILTKKKGSTQPGGVIGSIIHYPKDMTETAAEINGYRSLEFKANE

HGPI

>Teni-HAA *Tetraodon nigroviridis* (tetraodon). CAG07665.1

MHFFGILLLLFLEPLEGFHVPVDAVIVQVQSWGAVGAQQLRGHVLLNGAALTGENQEVDA

IIRSMSVHQHLPSAIGVNLTSVLRNCTLLRSRECILEGSELHWTDRVFCDGKVYLTLDGN

DTWTPHVPQALALKESWDQQVERTREQRIRLQEGCFKLMGKLGLSAETSVAPVALLPQYV

IPVLCVLVFIGLIIISIVFSKTPGVIGSVIHYPQDVNEMVTEEKGRIYRTLWSEDPAL

>Teni-HAA T*etraodon nigroviridis* CR638748.2 full-length cDNA

MHFFGILLLLFLEPLEGFHVPVDAVIVQVQSWGAVGAQQLRGHVLLNGAALTGENQEVDA

IIRSMSVHQHLPSAIGVNLTSVLRNCTLLRSRECILEGSELHWTDRVFCDGKVYLTLDGN

DTWTPHVPQALALKESWDQQVERTREQRIRLQEGCFKLMGKLGLSAETSVASVALLPQYV

IPVLCVLVFIGLIIISIVFSKTPG

>Lacr-HAA *Larimichthys crocea (*yellow croaker). XP_010741942.1

MHCLGILVMLWDLSLAFTAPVDTIIVQVQQWGVVGAQRVVDQVLLNGVSLTDTSQEVDNI

VQAISADALLPTLISVNQSLLPKNHTVLRSRECILEGSQLHWTDRVFCDGEVYLTLDHSD

MWTAHVPQALAIKALWDQEVQRTKTERIRLEEGCVKLMRELRLSEVQSAQGIPLPQFLIP

VMALVAFIGLVLISLVLSKGYGLRHPGGVIGSVIHYPKDMTDMPQEIKGNGYRTL

>Lacr-HAA *Larimichthys polyactis* (redlip croaker). TSA: GETG01010010.1

MHCLGILVMLLDLSLAFTAPVDTIIVQVQQWGVVGAQRVVDQVLLNGVSLTDASQEVDNI

VQAISADALLPTLISVNQSLLPKNHTVLRSRECILEGSQLHWTDRVFCDGEVYLTLDHSD

MWTAHVPQALAIKALWDQEVQRTKTERIRLEEGCVKLMRELRLSEVQSAQGIPLPQFLIP

VMALVAFIGLVLISLVLSKGYGLRHPGGVIGSVIHYPKDMTDMPQEIKGNGYRTL

>Pore-HAA *Poecilia reticulata* (guppy). XP_008432358.1

MRFFTFVFIFFKHLSTALPLHVDAIIVQIQQWGVVGAQHVXEQVLLNGISLSSPSWEVQS

IIKSMSASDLLPDGLSNNQTSVLRNHTVLRSRECIIEGSRLHWSDWVFCDGKVFLTLEHN

DTWTAHAPEARALKNLWDQEVEHSKFERRRLQEGCIQLMKELKLSEEESVHGIALPRFLI

PILAVMAFLMLIIVTILISRGPGLIHPGGVIGSIIHYPKDVTHTDEKECGYRAL

>Pore-HAA *Poecilia reticulata* (guppy). TSA: GFHH01045885.1

MRFFTFVFIFFKHLSTALPLHVDAIIVQIQQWGVVGAQHVAEQVLLNGISLSSPSWEVQS

IIKSMSASDLLPDGLSNNQTSVLRNHTVLRSRECIIEGSRLHWSDWVFCDGKVFLTLEHN

DTWTAHAPEARALKNLWDQEVEHSKFERRRLQEGCIQLMKELKLSEEESVHGIALPRFLI

PILAVMAFLMLIIVTILISRGPGLIHPGGVIGSIIHYPKDVTHTDEKECGYRAL

>Orla-HAA *Oryzias latipes* (medaka). Chr.17:25.554.366-25.555.754 FGENESH and DK062931.1

MRITELQFCSFIYFLLSQLSQGDPVLPPADAIIIQIQHSGVVGGNYVAERVLLNGVPLSS

TSQEVSNILQSLSNKELLLFSASISQMPALTEGHIIVCHQECILEGHQLHWTDRVFYNGT

VYLSLEPNGTWTALVAQAMVLKGILEPNGHHTESESTRLQNGCMQLMKELKFSDKPSGSG

IFFIQIIIPVFVVFAVMFTLSILVSRSQGWTYPGGVIASIIHYPKNLSNTDPYTNDYGYS

TL

>Nofu-HAA *Nothobranchius furzeri* (turquoise killifish). JZ213307

MFHETGLDSKWRKMHLYEVVMLALFHFSSEAVPLPVEAIIVQIQQRGVVGDQHVTEHVLL

NGVSLRSPTQEVKNILKTLSAGPLLPATTTFNQISALKNHTIIRSRDCIKEGTLFHFSDH

VFCDGKVCLTLEHNNTWTAHIPQAEALKVVLDQQLTGAEGTSLWEGCIQLMKELKLSGEQ

SVSPFALNPVLVPVLAILTFFGLILLSMFVSNKLGSRHPGGVIGSVIHYPQDVDTVELKS

RGYSLL

**Protacanthopterygii** (Salmonidae/ Esociformes)

>Sasa-HAA *Salmo salar* (Atlantic salmon). XP_013995094.1 and TSA: GEGX01039681

MRLPLTFILFFKLNWGLPIDTVILFQRSGVVGEMDTVEQVLVNGAPLSSCGKYVSGILRA

VLLGNDSESFQQAFEMEENVTNTITKNPTYQYLRVRECKLNGFRVVHLTDQLLVNGSDFL

TLDQSTVTWTAEVPQALALKQLWDWDTERTRRERMQLHESCTELMKELTHSEPTTNRAGM

SLLTVLAPLLASLAFVAVVIASFLIANRQDPRVSGHPGGVLGSIVHYPQNVTETPQAPQS

GKDYQVL

>Sasa-HBAψ *Salmo salar* (Atlantic salmon). Ssa03_NC_027302.1:17030685-17037352, internal stop codon, pseudogene

VDMVIQFQRSGVVGEVKTVEQVLISGAALSSSEKDVSGILRAVLLGNYSESFQQAFEIEE

NVNNTMTENHTYQYLRVHESKLDGFQVVHLTDQLLVDRNDFLTLDRNTDTWTAEVPQALA

LKQL*DQDTERTRLDRIQLHESCAKLMKEITHSEPTTPAGVLGSIVHYPQNVTETPRPGK

GYRVL

>Onmy-HAA *Oncorhynchus mykiss* (rainbow trout). Chr. 8 NC_035084.1: 52.835.011-52.852.788 XP_021468778.1

MQLPLTFILFFNLSWGLPIDTVILFQRSGVVGEMETVEQVLVNGAPLSGSGKYVSGILRA

VLMGNYESFQQAFEIEENVTNTMTKNPTYQYLRVRECKLNGFRVVHSTDQLLVNGNDFLT

LDQSTDTWTAEVPQALSLKQLWDWDTEFTRREKMQLHESCIELMKELRHSEPTTNRGTSL

LTVLAPLFACLTFVVMVIASFLISKQRDPRVSRHPGGVLGSIVHYYQNVTETQSGKDYQA

L

>Onmy-HAA *Oncorhynchus mykiss (*rainbow trout). CU063683.1

MQLPLTFILFFNLSWGLPIDTVILFQRSGVVGEMETVEQVLVNGAPLSGSGKYVSILRAV

LMGNYESFQQAFETEENVTNTMTKNPTYQYLRVRECKLNGFRVVHSTDQLLVNGNDFLTL

DQSTDTWTAEVPQALSLKQLWDWDTEFTRREKMQLHESCTELMKELRHSEPTTNRGTSLL

TVLAPLFACLTFVVMVIASFLISKQRDPRVSRHPGGVLGSIVHYYQNVTETQSGKDYQA

L

>Onmy-HBAψ *Oncorhynchus mykiss* (rainbow trout). Chr.28. NC_035104.1: 26.645.363-26.651.156, pseudogene

MRLPLTFILICELCWGFKRDMVIQFQRSGVVGEVEQVLINGAALSSCEKDVNGILRAVLL

GNYSESFQQAFEIEENVNNTMTHESKLDGFPVVHLTDRLLVDGNDFLTLDRNTDTWTAEV

PQALALKHLWDQDTERTRLERIQLHESCAKLMKELTHSEPITTGGNAYLVTLCPAGVLGS

IVHYPQNVTETPCPGKGYQVL

>Onki-HAA LG30 *Oncorhynchus kisutch* (coho salmon). NC_034203:15.300.093-15.319.385 XP_020322949.1

MLLPLTFILFFNISWGLPIDTVILFQRSGVVGEMETVEQVLVNGAPPSSGGKNVSGILRA

VLMGNYESFQQAFEIEENVSNTMTKNPTYQYLRVRECKLNGFRVVHSTDQLLVNGNYFLT

LDQSTDTWTAEVPQALSLKRLWDWDTEFTRREKMQLHESCTELMKELRHSEPTTNRGTSL

LTVLASLFACLTFVVMVIASFLMSKQQDPRVSRHPGGVLGSIVHYYQNVTETQGGKDY

>Onki-HAA *Oncorhynchus kisutch* (coho salmon). TSA: GDQG01022517.1

MLLPLTFILFFNISWGLPIDTVILFQRSGVVGEMETVEQVLVNGAPPSSGGKNVSGILRA

VLMGNYESFQQAFEIEENVSNTMTKNPTYQYLRVRECKLNGFRVVHSTDQLLVNGNYFLT

LDQSTDTWTAEVPQALSLKRLWDWDTEFTRREKMQLHESCTELMKELRHSEPTTNRGTSL

LTVLASLFACLTFVVMVIASFLMSKQQDPRVSRHPGGVLGSIVHYYQNVTETQGSCCLDM

>onkiHAAψ NC_034203.1: 15290463-15297915 (rev) pseudogene lacks 5’ end

ENPTYQYCVCCECKLNGFRVVHSTDQLLVNGNYFLTLDQSTDTWTAEVPQALSLKRLW

DWDTEFTRREKMQLHESCTDLMKELRHSEPTTNRGGTSLLTVLASLFACLTFVVMVIA

SFLMSKQQPAGVLGSIVHYYQNVTETQGG

>onkiHBA LG 27 *Oncorhynchus kisutch* (coho salmon). NC_034200:25424828-25439103 XP_020319115.1

MRLPLTFILICELCWGFKRDMVIQFQRSGVVGEVEQVLINGAALSSCEKDVNGILRAVLL

GNYSESFQQAFEIEENVNNTMTKNHTYQYLRVHESKLDGFRVVHLTDRLLVDGNDFLTLD

RNTDTWTAEVPQALALKHLWDQDTERTRLERIQLHESCAKRMKELTHSEPITTGGGMSML

AALASLLAGLALVAMVTTSFLIFKQQDPNVSGHPGGVLGSIVHYPQNVTEKPCPGKGYQV

L

>Eslu-HAA *Esox lucius* (northern pike). XP_010881869

MIANMQLPLITILLFKSCLCLQIDTVIQFLRSGVFGKVETVEQVLVNGAPLSSCGKDVSS

ILNAVLLINYNESFQTIEESGNNTMKISHTYMRVRECKLDSFGVLLLTDRLLVDGNNFLT

LDRATDTWTAEGPQALPLKHLWDRESTRTRREIMQLHESCTKLLEEITCSEPGATGAGMD

IPTALAPLLAGLGFVILVIASFLISKKQDPTISGHPGGVIGSIIHYPHNISELSHSPNPG

RGYQVL

**Cypriniformes**

>Cyca-HAA *Cyrpinus carpio* (common carp). Chr.4:5.071.107-5.074.000 KTG41314

MLLFATFAFLVDLSWALPTDIIIQFERSVLLNGTVVERILVSDANLKGHFDKQPVVRPIQ

DEVLKTMVENDHETSSYGTYIRLRECKLRGYRVLQLSDRFQLNGKDYLTLDSDRDSWTAL

MPEAHDLKKSWTLKAEHASLEKTHLKEECEEFVKQINDAQNQEGLGVLRVMAPVLATFLF

IVFVFMSLLIFKRHGHHPGGVLGSIIHYPAHHLEAPLNNKQSQKDISQVPMLKVPNSNHL

TCPTTLL

>Cyca-HBA *Cyrpinus carpio* (common carp). Chr.4:4.975.004-4.977.896 KTG33590

MLLFFVSFAFLVDLSWALPTDIIIQFERSVLLNGTVVERILVSDARLKGQFDKQPVVRPI

QDEVLQMMVENYHATASYGTYIRLRECKLRGDRVLQLSDRIQLNGKDYLTLDSDTDSWTV

LMPEAQDLKKSWTLKAEQASLEKIHLKQECEESIKQWNDTQNQEGLGVLRVIFPVLATIL

FIGLVLISFLIFKRHGHQPGGVLGSIIHYPAHHLEVPLDRQSQKNIAQVPVLKGP

>Cyca-HAA *Cyprinus carpio* (common carp). TSA: GFWU01041615.1 SVLLNGTVVERILVSDANLKGHFDKQPVVRPIQDEVLKTMVENDHETSSYGTYIRLRECK

LRGYRVLQLSDRFQLNGKDYLTLDSDRDSWTALMPEAHDLKKSWTLKAEHASLEKTHLKE

ECEEFVKQINDAQNQEGLGVLRVMAPVLATFLFTVFVFMSLLIFKRHGHHPGGVLGSIIH

YPAHHLEAPLNNKQSQKDISQVPMLKVPNSNHLTCPTTLL

>Cyca-HBA *Cyprinus carpio* (common carp). TSA: GFWU01041655.1, Internal Stop codon

TDIIIQFERSVLLNGTVVERILVSDARLKGQFDKQPVVRPIQDEVLQMMVENYHATASYG

TYIRLRECKLRGDRVLQLSDRIQLNGKDY*LTLDSDTDSWTVLMPEAQDLKKSWTLKAEQ

ASLEKIHLKQECEESIKQWNDTQNQEGLGVLRVIFPVLATILFIGLVLISFLIFKRHGHQ

PGGVLGSIIHYPAHHLEVPLDRQSQKNIAQVPVLKGP

>Dare-HAA *Danio rerio (z*ebrafish). XM_003197841

MLLMVTFAFLVALSWAFPTDLVIQFERSVVNGTVVERIFVNDAHLKGHQPVVRPIQDEVL

KTMVENSHKTAGYATYIRLRECKFRGYQVLQLSDRFQLDGRDYLILDPQTDSWIMQMPEL

HDLKQPSMPEAESTSLEKIHLKDECEELIKEMNDTQNQEGFDVLRVIAPVLSMFFFVGFI

FISFLMFKRHGQHPGGVLGSIVHYPPFHTVPSDGQSQKNIDQVPVLKCPH

>Dare-HAA *Danio rerio* (zebrafish). TSA: GFIL01014849.1

MLLMVTFAFLVALSWAFPTDLVIQFERSVVNGTVVERIFVNDAHLKGHQPVVRPIQDEVL

KTMVENSHKTAGYATYIRLRECKFRGYQVLQLSDRFQLNGRDYLILDPQTDSWIMQMPEL

HDLKQPSMPEAESTSLEKIHLKDECEELIKEMNDTQNQEGFDVLRVIAPVLSMFFFVGFI

FISFLMFKRHGQHPGGVLGSIVHYPPFHTVPSDGQSQKNIDQVPVLKCPH

>Sirh-HAA *Sinocyclocheilus rhinocerous* (horned golden-line barbel). NW_015649561.1:87.947-91.348

MLLFATFAFLVDLSWALPTDIIIQFERSVLPNGTVVERILVSDANLKGQFDKQPVVRPIQ

DEVLKMMVENDHETASGYGTYIRLRECKLRGYRVLQLSDRFQLNGKDYLTLDSDSDSWTV

LMPEAHDLKQSWTLKAEHASLEKIHLKEECEEFIKQINDAQNQEGGLGVLRVMAPVLATF

LFIGFVFMSLLIFKRHGEHTTGIMGVLGSIIHYPAHHLEVPLNEQSQKDISQVPMLKA

>Sirh-HBA *Sinocyclocheilus rhinocerous* (horned golden-line barbel). NW_015666593.1: 418.897-426.574. Internal stop codon

LLFFVTFAFLVDLSWALPTDVIIQFERSVLVNGTVVERILVNDAHLKGQFDKQPVVRPIQ

DEVLKMMVENDHRVTASGYGTYIRLRECKLRGYRVLQLSDRFQLNGKDYLTLDSDTD*WT

VLMPEAQDLKQSWTLKAEQASLEKIHLKEECEEFIKQRNDTQNQEGGLGVLRVLAPVLAT FLFVGFVLMSLLIFKRHGEHKTGIMGVLGSIIHYPAHHLEVPLDGQSQKNIS

>Sirh-HCA *Sinocyclocheilus rhinocerous* (horned golden-line barbel). NW_015662004.1:1.803.008-1.803.959

MLLFATFAFLVDLSWALPTDIIIQFERSVLPNGTVVERILVSDANLKGQFDKQPVVRPIQ

DEVLKMMVENDHETASGYGTYIRLRECKLRGYRVLQLSDRFQLNGKDYLTLKDN

**Characiformes**

*>Asme-HAA Astyanax mexicanus* Mexican tetra. LC494124 and LC494125. 3’end sequence was not found in the genome.

MVSAEMLWMMMLVFLGGSPLWAFPLAPPPGVVECEYAGRRVELLRDRVRVEGRDVLVLNQ

VNHTWTMLVTEPAQLDLQQILQECTDLKKKMLNYNQTTTGVSVFGVVATLLAALLFVGFV

LLSFKYPEYVGVGGVLGSIIHYPPHSQKEKEKGRSENVPPANVSPY

*>*Coma-HAA *Colossoma macropomum* (tambaqui). TSA: GGHL01056846 and overlapping SRAs of Bioproject PRJNA292457.

NPTYVRECVYEGHWVVLLSDRLQVDGKDVVVLDKVNHKWTVFSADGHQDPELTNLDTLQIL

EGCEELKKELSHMENSAGVSWSRVVSIVLATLFFISLVVLSFKMSKRYGAAGGVLGSIIHY

PAHFPKDQSEKRFSHLPTYLLTTKDTVSR

*>*Pime-HAA *Piaractus mesopotamicus* (pacu). Overlapping SRAs of Bioproject PRJEB6656.

MTPLLLRCLTLLFLWSLCAARPTENPTYISECLYKGHRVVLLSDRLQVDGKDVLVLDKVNH

KWTRADGHQDPALTNLDTLQILEECEQLKKEFSRMENSAGVSWSRVAFIVLAVLFFISFVV

LSFKMSKRYGAAGGVLGSIIHYPAHFLKDQNEKRFS

**Siluriformes**

>Icpu-HAA Ictalurus punctatus (channel catfish). NC_030435.1: 21.975.148-21.977.640

MKMKMKMMLLFLSVLLFLSQFSAGCVCEPVENAVNTTRLINTLWIIRNKPTSPVNLTCVCL

CECRRVVQLNINDSAIEVKESEREMLEDECVKLVKDLSTKKESRDLSVLSVATPLVGASVF

ITLLLLSFAMFKRQGVLGSLIHYPAHSLERTTNPGAFLPPETKIPY

*>*Icpu-HAA *Ictalurus punctatus* (channel catfish). TSA: JT437950

MKMKMKMMLLFLSVLLFLSQFSATFPLKGCVCEPVENAVNTTRLINTLWIIRNKPTSPVNL

TCVCLCECRRVVQLNINDSAIEVKESEREMLEDECVKLVKDLSTKKESRDLSVLSVATPLV

GASVFITLLLLSFAMFKRQESQAGGVLGSLIHYPAHSLERTTNPGAFLPPETKIPY

*>*Icfu-HAA *Ictalurus furcatus* (blue catfish). Overlapping SRAs of Bioproject PRJNA195453. MKMKMKMKMKMKMMLLFLSVLLFLSHFSATFPLKGCVCEPVENAVNTTRLINTLWIIRNKP

TSSVNLTCVCVCECRRVVQLNINDSVIEVKESEREVLEDECVKLVEDLSRKKESGDLSVLS

VVTPLVGASIFITLLLLSFTMFKRQESQAGGVLGSLIHYPAHSLEKTTNPGTFLPPETKIP

Y

*>*Sime-HAA *Silurus meridionalis* (Southern catfish). Overlapping SRAs of Bioproject PRJNA427243.

MMMMVLFISALLFLSHFSASFPLKGFVYEPGENPTNHTSTTHLPGVLRMTQNTPTSSSGEC

GFSPIALLVSVFISLLLLSIWMLATQESHPGGVLGSLIHYPAHSLENTMKKGTTSRD

*>*Sias-HAA *Silurus asotus* (Amur catfish). TSA: GHGF01004423.

MQKKVKKMMVLFVSVLLFLSHFSASFPLKGFVYDPGENPTNPTSTTHLPDVLRMTQNTPTS

SSGESGFSPISLLVSVFIALLLLSIWMLATQESQPGGVLGSLIHYPAHSLENTMKSPETKL

PH

**Gymnotiformes**

>Eivi-HAA *Eigenmannia virescens* (glass knifefish). TSA: GGGZ01064726

MLLLITIAFFGNLSCGLPDGPTGVVKDKWATKEVVLVNDVSVGLLHKDVHSMIAISRSFDA

LTTLVNSKLFPNNLTNVNFHECNFKGQQSDRPGKDILVLYPVNYTWAVLIPQTGDISEIWN

KETARSVLEGRQLEEECATILQKVARLDDAPGINTVSVVGVILATIILVTLILLSFVMFKY

QVAHVGGVLGSIIHYPAHSLDASLRDTNNQLELSDRPPVT

**Clupeomorpha**

>Clha_HAA *Clupea harengus* (Atlantic herring). NW_012220971.1: 1.071.227-

1.073.474

MRMLRTLYFLCMVTLSAGLPMGTVIRYEYNSVFGEEETFQERIFINGSPVTPNGNGDVRT

GVIDVMSFNNGSSSLHDSLRVFANSFNKTKKDHTYQRLRECKFNGHQVMHISDRIKHNGK

DYLSLDQTTDTWVALDPQAVFLKKELDRDSERTVRDRMCFQEACTELMKDLNHSYSASKG

GSATVLAPLLAVLILFGLVILSVMSKHRVLGSIVHYPAQTSDTVKEPAIQPSLSIKASLL

SNMP

*>Teil-HAA Tenualosa ilisha* (hilsa ilisa). QYSC01123722.1: 356.174-357.933

MQILCTIYFVGVIALSTELPVIQLEYKSVISEEGTVEQIFVNGVSLTLDGRTEAFNVMRP

GNGISATLRDALRIFANRFNNTDNHTYQRCRKCRFNGHQLIHVSDQIMHNGKDFLSLDQM

TDAWTALDPQAFFLKEKLDRDHESIMQERVRLQEACTELRKEFSHSQFAPRVSAEVLIPL

LAALVMLVLILLSLIISKHGVLGSIIHYPGPTSDIIREPVKQPSHSIKACLLSNMS

*>Sapi-HAA Sardina pilchardus* (sardine). TSA: GGSC01229082

MWMLHTFYFVCLTALSAGLPDTSINFVRLEFRILISKQQTFEENIYIYYGTSGTPNGSRD

GRPASLSNSSSSLRDALLIFTNRFNNTENHTYKRCRECKFIGHRLIHVSDQIMHNGKEFL

SLNQTTDTWTALDRQALSLKEALNRDSERTVQDRMRFQETCAELLKELTQSHSALEVSAA

VLAPLLAGLLLFGMVLLSCILAKQGVKGSAHPAGGVLGSIGHYPAPTSDVVREPILQPSL

SKAHLLSNMP

>Alal-HAA *Alosa alosa* (allis shad). TSA: GETY01043622 MWMLHTFYFVCLIALSEELPDKSTISFVRLEYKSVISEEQTFEEKILINGTSVTPNDNGD

GRATEIYRTLLGNISSSSLRDALLIFANRFNNTENHTYQRFRECKFIGHQLIHVSEQIMH

NGKEFLSLDQTTDTWTALDPQALSLKEVLDRYSERTVQDRMRFQETCAELLKELTHSDSA

SKVSTAVLAPLLAGLVFLGLVLLSFIMSKHSVKGSAQPAGGIVGSIVHYPAATSDIVREP

TLKPSLSIKAHLLSNMP

**Elopomorpha**

>Anan-HAA *Anguilla anguilla* (European eel). TSA: GFIC01029617.1

MGIQIIFSLLWTVFFSLHEAKETTIRYQHSGIVGGSPTSETLTIDSVPIGWSDRRNNGFL

SINTECQGLFRMTLRMITRSFNHTTTNNHTYQRTHECFLEGHRVLRVSDRIHYDGAEYLC

LDSAKNTWMAAVPQALALKQQWDRESGCTERHKVLLQNGCTELIEEFSRNKHSAPEASLA

IVLIPILIIVVLLCQFMVSFLISRHGSRLAGGVLGSIIHYPQRNVGIKPLKGDPEEHNLA

FTPSKGVTLFPAGHPNLGRDTAGQCGATCGPNRAICKKLIEAFVDPCFAQN

>Anro-HAA *Anguilla rostrata* (American eel). LTYT01001052.1:54564-57749 FGenesh

MGIQIIFSLLWTVFFSLHEAKETTIRYQHSGIVGGSPTSETLTIDSVPIGWSDRRNNGFL

SINTECQGLFRMTLRMITRSFNHTTTNNHTYRRTHECFLEGHRVLRVSDRIHYDGAEYLC

LDSAKNTWMAAVPQALALKQQWDRESGCTERHKVLLQNGCTELIEEFSRDKHSAPEASLA

IVLIPILIIVVLLCQFMVSFLISRHGSSLAGGVLGSIIHYPQRN

**Holostei**

>Leoc-HAA *Lepisosteus oculatus* (spotted gar). XP_015216910

MRPFFIFIFFQTLTLPVTAGTFTLRYLYTGVARDVDRFSFSAVLTLNHVPILFYDSRTGN

IVPRQEWLREAFDSAFWDTERMRNREQLIVFESTMLLMMQSFNHTTAEPHTYQRLRECKL

EANGDVRVSDRFGYDGTDYLSLDQGTGTWIPAVPQAQSVKQKWDADTARTQECRVYLEEE

CIQVLKTLIRNGTGALERPVYVEVFQVEREPWEFCCLVTGISSSGAEVQWVVDQQGVLAE

GQSREELLPNGDGSFQVRQVLRVSQQERERHSYACQVNGTLQEPSFPKLRSKFAFFAFLL

TILALSVLVCAGLYYLSVVKTRTQGSDQLRYVPGPLGSTGFYHPGEQHNEP

>Leoc-HAA *Lepisosteus oculatus* (spotted gar). TSA: GFIM01030402.1

QTLTIPVTAGTFTLRYLYTGVARDVDRFSFSAVLTLNHVPILFYDSRTGNIVPRQEWLRE

AFDSAFWDTERMRNREQLIVFESTMLLMMQSFNHTTAEPHTYQRLRECKLEANGDVRVSD

RFGYDGTDYLSLDQGTGTWIPAVPQAQSVKQKWDADTARTQECRVYLEEECIQVLKTLIR

NGTGALERPVYVEVFQVEREPWEFCCLVTGISSSGAEVQWVVDQQGVLAEGQSREELLPN

GDGSFQVRQVLRVSQQERERHSYACQVNGTLQEPSFPKLRSKFAFFAFLLTILALSVLVC

AGLYYLSVVKTRTQGSDQLRYVPGPLGSTGFYHPGEQHNEP

>Amca-HAA *Amia calva* (bowfin). TSA: GEUG01019669

MSSFLMLCLLSAVGMPVTKGTFTLRYLYTGSIGHPDQFVALVTLNGTQIMSYDSGSGKTV

PQLDWLTEDLDVKFWSAENQRNMEQQLVFKRMSVAMLRSFNHTTVDGHKYTRVSECELDG

SKVSQLSDRFCYDGGDFLILDRFADTWRAAIPQAQGTIQMLDASTVRVDLEQECMVFRET

LTHLEGAPLAQQAPSSVTFTISVVLFTVMVCLGLIVISCVIAKKRGTNGPGYIAGPLGSL

GVYYAGNQKTNPDIV

**Other deduced MHC class I amino acid sequences used in this study:**

Z lineage sequences:

>Sasa-ZAAa *Salmo salar* (Atlantic salmon). Z lineage sequence. ACX35596.1 GQ505858.1

MNISHLTVFVLYFSLECICQSDTYSLSYIYTALSKPVDLPGIHEFTAMGLMNNQQIDYYD

SVSKKKIPKQDWMREKLPADYWEKGTQSRKSKEQWFKVNVDILMKRMRHNNTDVHVLQWK

VGCEIDQQSDGTLKFIKGIDQYSYDGDDFLAFDDVTMQWVAPVDQALPTKRKLDDVQILN

TYTKGYLEKECVDWLSKFMEYEDKEFSWADSAPKVYAFAKKAKTAGHVRLTCMATGFYPK

DVVMHIKKNGVPLTDRDGVQSAGLLPNDDETYQIRMSVQIPEADKETYECYVNHRALKEP

IVVKWDGKCCDCSSGGAVVIGAVVIAFIVVLILVGLFVLHRRGTIGRS

>Leoc-LO14 *Lepisosteus oculatus* (spotted gar). Z lineage sequence. TSA: GFIM01040660.1 and Grimholt et al. 2015.

MTRGPALLLVLALCWPASGERHSLHYIYTALSKPVDGIPEFTAMGVLNDRQIDYYDNWIM

QKIPKQSWMKTNMGQEYWEKGTQSRKSKEQWFKVNVGILMDRMRQNNSDLHILQWMHGCE

IEVQPGSKPQFLRGYDQYSYDGRDFLSFDESKMQWVAPVWPAEPTKHKWDNEQILNQYTK

GYLERECVDWLTKFLGFGEQQIKKSVPPDVHMFAKRSLTPGQLTLTCLATGFYPPDVQVD

LYRDGVMLLEKDRVLSSGVRPNGAEEDTYQLRKSLEIRETDKRSYSCEVKHSSLKEPVVR

QWERTVWVPPASPPSNLPIVVSVVLLVVALVAGGAALAFFAHRRKKASGSDSSGSPRSSD

SDLQTNGKTISNGNGVSVPLISNGGQTQPNGGLIPEGVPFVLPV

S lineage sequences:

>Sasa-SAA *Salmo salar* (Atlantic salmon). S lineage sequence. ACY30362.1

MITTILISFMQFSIVAPHSLHRHCIATQGTLYPKNIQLVMIDDVTVYYYNSSAEQEAVVP

EWLNHLEGIEFWQEVNRNLKFSRFVMDTAVRVTSEHYNHSHDHFYQAHGRCGWKSDGTTE

AFMSHAYDGKDFVSFDVSTRTWTAAVSHAVFYKRKRETDLEDLVRLVIHYESGCIRWLKK

LLQFSVTFREPKVPAVSLFERPPHGNSEVEVTCHVTGFYPRAVQVEWLGAEGLPMVDGVS

SGEVLPNGDGSYQLRKSLTVPQEAQDTQSYSCLVLHSSIAGNITVTWAPKKNLANVLMAI

VIIVSVVLILTVLFKYLVRRRAVGKSQS

>Asme-AM33 *Astyanax mexicanus* (Mexican tetra). ENSAMXP00000017 KB882192.1:1,855,138-1,867,212 (Grimholt et al. 2015)

MEKMKESYIHCVLLLLLLHLLQLSGGEVNSITAYFIGIQGLNLPDYMERIAVNDVTMFYY

DSSMKDEVSCPDWLNTSSGKQHWMDINLISLHNKHSMATALKSAILQFNQTASSSDVNIY

QGYGRCSVYPNGTLKALLTHAFNGKDFLTFDVDRKSYIASVPQAVIYKRQREANPVWLEI

MASFYKKTCFERLKMFLQHASVHITKKVPEVHLFKSFKSGSSVLACHVTGFYPKEVQVEW

IGAGLQPVDGEVIEVLPNGDGTYQTRRSVIRPEENPEKHSYSCVVQHSSIAGNITKTWVA

EEHSLLAVWISLVCILVIIGTGLVLRKFCRCGQRDTGI

L lineage sequences:

>Sasa-LCA *Salmo salar* (Atlantic salmon). L lineage sequence. XP_013983104.1. (Grimholt et al. 2015)

MGKLSVFLFVLSFYTIVNSGSGSHSLWALATYISGETPFPEFTVVVMLDDVQVAYYDSNM

KHFIYRGHNTPNKIHDDEAKNGDFVFGVMYHHMKERYFHLKHHLNLTEGVQVQQRMAGCE

MFDNGEPALIMTKNTFNAVFADHAIYYNITHFTYDAGKLLQGWDGMRQAQEKILYENVLL

TLCIRTLKTILKREKNIVMRKVPPRLRLIKKEVSGGFQVSCLVFGFYPRHINLTLLRDGQ

PVAEQELTGGEVLPSGDGTYQLRKSLEVSTEELKKRHNYTCTASHLSLDNKLDVSWESGA

ERVHLSTLSVLLVMLLILILLVTFICVKRRWSNTASQSELANVDAKVSEEMNLSSDSEN

>Leoc-LO12 *Lepisosteus oculatus* (spotted gar). L lineage sequence. JH591577:52,184-56,541 (Grimholt et al. 2015)

MKPSALFLLCCGELAWADSHSLWYFMTLTTGPSQFPEFVVVGMVDDVQVEYYDSDVGKVI

SRRHWRPDAEVEEADSKVTAAKHYHNSMRNKLQLLMSHLNHTGGHRTYQRIAGCELDDDG

SARFGRWDAYDGRDALVYNTQSYSWSLLIPQVVIDKALFQVNKIRADTFYQPLCVRVLKS

YLQQERTRLMRRVKPRVRVFQKTSAFSGGTEVTCLATGFYPRALELTLLRDGRPVPEQEL

TGGEVLPNGDGTYQLRKSLALSEEEEERRGRHRYTCRVQHSGLDNGLEVDWEPEPDLDTG

LIVGVVIVVLIVVLVLPVAACVLWRKK

P lineage sequences:

>Taru-TR6 *Takifugu rubripes* (fugu). P lineage sequence. Scaffold_497:44,782-49,340 (Grimholt et al. 2015)

MNIGLLFWILLPAAECGSHSLEFLSTGRVQPGSGPHFEQVTEFDGVVISHCDSGTQQEHF

KPVLESHNLPGTCRPACYDVFDALKEISKFINHTRNVQRRRGCITSDERLESAFDNWAVN

GEGFIQFDAGAQKWKALSPSAEMIKDSWNGYEAQNHVFGQFIRRYCPEMIHQIKLRETEK

RTDLRVFANPVDLTKALLKCHVTSTDKSVRSVSLTEDGATKANWITVTGPLPSGDGSVIL

ILTAEVPLIHTNIYGCVVQTEDRNITVMWDGNTLDGRHILYAGVPMKFWIIIGIIFVCCL

ISVMTLLCKSHFFDCNFQVSL

>Asme-P *Astyanax mexicanus* (Mexican tetra). P lineage sequence. TSA: GFIF01000014

MIFILLCVYCSVLETTSAEKHTLQYLYTLRSAPEDDTEFEITTVFDGLIISHCKSPRFRD

QSRYDWISQTFTDAEWKNRDLFCESEYYFHKTLKKKIEGVYNTTNGIIQRERSCTDSEDD

SVVLMSDSWGVNGEDFLTLDPKTLKWSSDTPLATPVQSDWNQMKFMTPSLMDFKMNQCKP

SLMKLKKKKEEYLKGNLRPKIYIFGKPSHDGDAVSLRCYVSHKYLSGVRVRLTLDGVVVD

NVNISSPAPNMDGSVQIRLETETNIKEPNRYHCVVDADNLHIFTAWDGQTLNRKPASRIY

EAWIPFILVSVLIIIACIMSGTFNGCFICCNKVLIQRNTNPSTEVNFLLFVVSEEKFRHE

AEVDDEFHGPIRPRVRSL

U lineage sequences:

>Sasa-UBA *Salmo salar* (Atlantic salmon). U lineage sequence. UBA*0301 XP_014032819 AAN75116.1

MKCFILLLLGIALHSSSAATHSLRYVYTATSGIPDFPEFVTVGLVNGEPISYYDSIIRRE

TPRQDWMAKTEGSDYWESQTQVSIGSEQTFKANIDVAKQRFNQTGGVHVNQKMYGCEWDD

ETGVTEGFDQDGYDGEDFLAFDLKTLTWIAPTPQAVITKLKWDSNTAQNEYRKNYLTQTC

IEWLKKYLDYGKSTLMRTVPPSVSLLQKTPSSPVTCHATGFYPSGVMVSWQKDGQDHHED

VEHGETLQNDDGTFQKSSHLTVTPEEWKNNKYQCVVQVTGLQEDFIKVLTESEIKTNWND

PNIVLIIGVVVALLLVVVAVVVGVVIWKKKSKKGFVPASTSDTDSDNSGRAAQMT

>Leoc-U *Lepisosteus oculatus* (spotted gar). U Lineage sequence. TSA: GFIM01032149

MFRVLVLIICGVQAASAVTHSLRYFYTGVGGVSGFPEYTAVGLVDGQEFVHYDSDIKWMI

SKTEWIERNEGKDYWDRETQKQIGASQTFKANIGILKERFNQTGGVHTVQYMYGCEWDEE

SGATGGFRQYGYDGKDWLMYDLKTFSWVAPVQQAEITKRKWEADKADMEYLKTYLTQICIE

WLKKYVNYGRETLNRRERPQVSVFHKDSRSGSTELTCLATGFFPRDILMSWWRDGQELHED

VDSGEVLPNGDGSFQVRKSLRVRAGEEDKYSCRVDHKSLSPGEDIVRTWEPKSGFPIGIIV

GVVVGVLVLIAVVVGIVIWRKRQRGYDKASDSDSDSKSSNNPPAKA

Other sequence:

>HLA-A2 *Homo sapiens* (human). AAA76608.2

MAVMAPRTLVLLLSGALALTQTWAGSHSMRYFFTSVSRPGRGEPRFIAVGYVDDTQFVRF

DSDAASQRMEPRAPWIEQEGPEYWDGETRKVKAHSQTHRVDLGTLRGYYNQSEAGSHTVQ

RMYGCDVGSDWRFLRGYHQYAYDGKDYIALKEDLRSWTAADMAAQTTKHKWEAAHVAEQL

RAYLEGTCVEWLRRYLENGKETLQRTDAPKTHMTHHAVSDHEATLRCWALSFYPAEITLT

WQRDGEDQTQDTELVETRPAGDGTFQKWAAVVVPSGQEQRYTCHVQHEGLPKPLTLRWEP

SSQPTIPIVGIIAGLVLFGAVITGAVVAAVMWRRKSSDRKGGSYSQAASSDSAQGSDVSL

TACKV

**Reference:**

Grimholt, U.; Tsukamoto, K.; Azuma, T.; Leong, J.; Koop, B.F.; Dijkstra, J.M. A comprehensive analysis of teleost MHC class I sequences. *BMC evolutionary biology* **2015**, *15*, doi:10.1186/s12862-015-0309-1.
