# Supplementary material for: Discovery of a Novel MHC Class I Lineage in Teleost Fish which Shows Unprecedented Levels of Ectodomain Deterioration while Possessing an Impressive Cytoplasmic Tail Motif
Source: Cells. 2019 Sep 9;8(9):1056. doi: 10.3390/cells8091056 (PMC6769792; doi:10.3390/cells8091056)
Supplement: Supplementary file 1 [file cells-08-01056-s001.zip › Supplementary Text S2. Mexican tetra HAA RT-PCR.docx]

**Supplementary text S2: Amplification of Mexican tetra *HAA* by RT-PCR**

Total RNA samples of gill (sample 1) and a mix of internal organs (sample 2) of a Mexican tetra were converted to cDNA and then subjected to PCR experiments. The primers used for PCR amplification were *Asme-HAA 5’UTR.F1* and *Asme-HAA 3’UTR.R1* designed at the 5’ and 3’ untranslated regions of *Asme-HAA*, respectively, as indicated by the Transcriptome Shotgun Assembly (TSA) sequence report GFIF01006274. For either sample, the only clear band that was amplified was 659 bp as expected from the GFIF01006274 report, and no longer bands were detected (A). Sequence analysis revealed that these bands contained two, presumably allelic sequences, which only show one silent nucleotide difference in the *Asme-HAA* coding sequence and were named *Asme-HAA**01 and *Asme-HAA**02 [for their alignment see (B)]. These sequences were deposited in GenBank as accessions LC494124 and LC494125. The amino acid sequence encoded by these sequences is shown in main text Fig. 4. The experiment confirms that *Asme-HAA* lacks an α1 exon sequence, as also indicated by TSA report GFIF01006274 and by genomic sequence information (see main text Fig. 3).

(A) *Agarose gel electrophoresis of RT-PCR amplified Asme-HAA fragments*


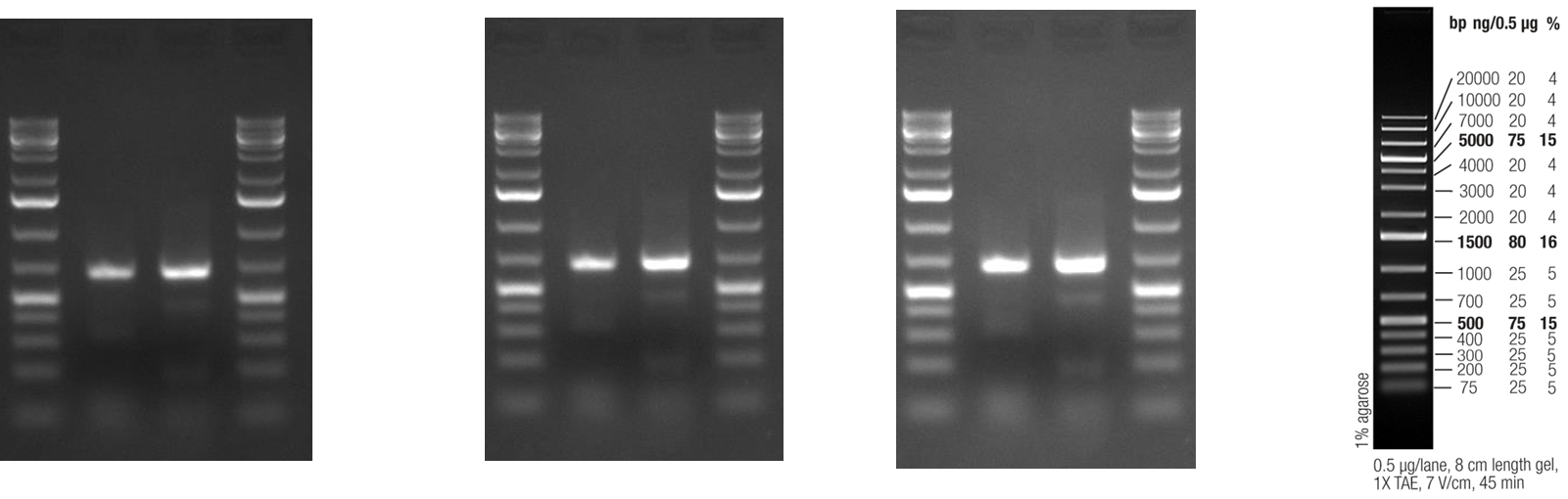
 1 2

700 bp -

1000 bp -

500 bp -

(B) *Alignment of the Asme-HAA*01 and *02 nucleotide sequences, with encoded amino acids above the second nucleotides of codons*

  M  V  S  A  E  M  L  W  M  M  M  L  V  F  L  G  G  S  P  L  
*Asme-HAA**01 1 ATGGTTTCTGCAGAGATGCTGTGGATGATGATGCTGGTGTTTCTGGGTGGTTCCCCACTC 60

||||||||||||||||||||||||||||||||||||||||||||||||||||||||||||

*Asme-HAA**02 1 ATGGTTTCTGCAGAGATGCTGTGGATGATGATGCTGGTGTTTCTGGGTGGTTCCCCACTC 60

 W A F P L A P P P G V V E C E Y A G R R  
*Asme-HAA**01 61 TGGGCTTTTCCTTTAGCTCCGCCCCCGGGCGTGGTTGAATGTGAGTATGCTGGTCGGCGG 120

||||||||||||||||||||||||||||||||||||||||||||||||||||||||||||

*Asme-HAA**02 61 TGGGCTTTTCCTTTAGCTCCGCCCCCGGGCGTGGTTGAATGTGAGTATGCTGGTCGGCGG 120

 V E L L R D R V R V E G R D V L V L N Q  
*Asme-HAA**01 121 GTGGAGCTGCTGAGGGACCGGGTTCGGGTGGAGGGTCGGGATGTCCTGGTGCTGAATCAG 180

||||||||||||||||||||||||||||||||||||||||||||||||||||||||||||

*Asme-HAA**02 121 GTGGAGCTGCTGAGGGACCGGGTTCGGGTGGAGGGTCGGGATGTCCTGGTGCTGAATCAG 180

  V N H T W T M L V T E P A Q L D L Q Q I  
*Asme-HAA**01 181 GTAAATCACACCTGGACGATGTTGGTAACAGAACCGGCTCAGCTGGACCTGCAGCAGATC 240

||||||||||||||||||||||||||||||||||||||||||||||||||||||||||||

*Asme-HAA**02 181 GTAAATCACACCTGGACGATGTTGGTAACAGAACCGGCTCAGCTGGACCTGCAGCAGATC 240

 L Q E C T D L K K K M L N Y N Q T T T G  
*Asme-HAA**01 241 CTGCAGGAGTGTACAGACCTGAAGAAGAAGATGCTCAATTACAACCAGACTACAACAGGC 300

||||||||||||||||||||||||||||||||||||||||||||||||||||||||||||

*Asme-HAA**02 241 CTGCAGGAGTGTACAGACCTGAAGAAGAAGATGCTCAATTACAACCAGACTACAACAGGC 300

 V S V F G V V A T L L A A L L F V G F V  
*Asme-HAA**01 301 GTCTCTGTTTTTGGAGTGGTGGCGACGCTTCTGGCAGCTTTATTGTTCGTTGGTTTTGTT 360

||||||||||||||||||||||||||||||||||||||||||||||||||||||||||||

*Asme-HAA**02 301 GTCTCTGTTTTTGGAGTGGTGGCGACGCTTCTGGCAGCTTTATTGTTCGTTGGTTTTGTT 360

 L L S F K Y P E Y V G V G G V L G S I I  
*Asme-HAA**01 361 TTACTGAGCTTTAAGTATCCAGAATATGTCGGAGTCGGAGGTGTTTTGGGCTCCATCATC 420

||||||||||||||||||||||||||||||||||||||||||||||||||||||||||||

*Asme-HAA**02 361 TTACTGAGCTTTAAGTATCCAGAATATGTCGGAGTCGGAGGTGTTTTGGGCTCCATCATC 420

 H Y P P H S Q K E K E K G R S E N V P P  
*Asme-HAA**01 421 CACTATCCTCCTCATTCTCAGAAAGAAAAAGAAAAAGGACGGTCTGAAAACGTTCCTCCA 480

||||||||||||||||||||||||||||||||||||||||| ||||||||||||||||||

*Asme-HAA**02 421 CACTATCCTCCTCATTCTCAGAAAGAAAAAGAAAAAGGACGATCTGAAAACGTTCCTCCA 480

A N V S P Y *

*Asme-HAA**01 481 GCTAATGTCTCTCCTTACTGA 501

|||||||||||||||||||||

*Asme-HAA**02 481 GCTAATGTCTCTCCTTACTGA 501
