# Supplementary material for: Discovery of a Novel MHC Class I Lineage in Teleost Fish which Shows Unprecedented Levels of Ectodomain Deterioration while Possessing an Impressive Cytoplasmic Tail Motif
Source: Cells. 2019 Sep 9;8(9):1056. doi: 10.3390/cells8091056 (PMC6769792; doi:10.3390/cells8091056)
Supplement: Supplementary file 1 [file cells-08-01056-s001.zip › Supplementary Text S3. Substitution rates.docx]

**Supplementary Text S3: Synonymous versus non-synonymous nucleotide substitution rates in the teleost HAA cytoplasmic tail motif encoding sequence indicate purifying selection**

In (A) (page 2) the nucleotide sequences of teleost *HAA* sequences encoding the conserved cytoplasmic tail motif GV(I/L)GS(I/L/V)(I/V)HYP are aligned. In (B) (pages 3-8) the analysis of the rates for synonymous (ds) and non-synonymous (dn) substitutions of these combined sequences are shown, while in (C) (pages 9-10) such analysis is shown for only those sequences found in Characiformes (*Asme-HAA*, *Pime-HAA* and *Coma-HAA*), Siluriformes (*Icpu-HAA*, *Icfu-HAA*, *Sime-HAA* and *Sias-HAA*) and Gymnotiformes (*Eivi-HAA*) (C/S/G fish). All results indicate clearly higher ds than dn rates, which concludes purifying selection at the encoded amino acid level. Figure (A) shows that there are12 nucleotide substitutions in this *HAA* region in C/S/G fish, but only one different amino acid among the encoded sequences. For the HAA amino acid sequences encoded by the individual H genes see main text Fig. 4.

The characiform sequences *Asme-HAA*, *Pime-HAA* and *Coma-HAA* are sufficiently similar for being relatively confident about the proper alignment of their available coding sequences (full-length in case of *Asme-HAA* and *Pime-HAA*, partial in case of *Coma-HAA*), yet they are different enough for a statistically meaningful comparison between ds and dn substitution rates. In (D) (page 11), it is shown that in all three comparison between the (available part of the) full-length *HAA* coding sequences when aligned in agreement with main text Fig. 4, the ds/dn ratio is around 2, confirming purifying selection at the protein level.

(A) *Teleost HAA cytoplasmic tail motif encoding nucleotide sequences, with the amino acid sequences encoded by the nucleotide fragments of Characiformes/ Siluriformes/ Gymnotiformes shown above their block of aligned sequences (Asme-HAA to Eivi-HAA)*

Orni-HAA ggt gtc att ggc tca att gtc cat tac cac

GAAC-HAA ggt gtt att gga tct att ata cac tac ccc

Lacr-HAA ggt gtt att ggc tcc gtc ata cat tac cca

Pore-HAA ggg gtg atc ggc tcc att atc cat tac ccc

Teni-HAA gga gtt att ggt tca gtg ata cat tat ccc

Sasa-HAA ggt gta ctt ggc tcc att gtc cac tac cct

Onmy-HAA ggt gta ctt ggc tcc att gtc cac tac tat

Onki-HAA ggt gta ctt ggc tcc att gtc cac tac tat

Onki-HBA ggt gtc ctt ggc tcc atc gtc cac tat cct

Eslu-HAA ggg gtc att ggc tcc ata att cac tat cct

Cyca-HAA ggt gtt tta ggc tca att atc cac tat cca

Cyca-HAA ggt gtt tta ggc tca att atc cat tat cca

Sirh-HAA ggt gtt tta ggc tca att atc cac tat cca

Dare-HAA ggt gtt tta ggg tca att gtc cac tat cca

G V L G S I/L I H Y P

Asme-HAA ggt gtt ttg ggc tcc atc atc cac tat cct

Pime-HAA ggt gtt tta ggc tcc atc atc cac tat cct

Coma-HAA ggt gtt tta ggt tcc atc atc cac tat cct

Icpu-HAA ggt gtt tta ggc tct ctt att cat tat cca

Icfu-HAA ggt gtt tta ggc tct ctt att cat tat cca

Sime-HAA ggc gtt tta ggt tct ctc att cat tac cct

Sias-HAA ggt gtt tta ggt tct ctt att cat tac cct

Eivi-HAA ggt gtt ctt ggt tcc atc atc cac tac cct

Alal-HAA ggt att gtg ggt tca ata gtc cac tat cca

Clha-HAA ggg gtt ctg ggt tca ata gtc cac tat cca

Teil-HAA ggc gtt ctg ggt tct ata atc cac tat cca

Sapi-HAA ggt gtt ctg ggt tca ata ggc cac tat cca

Anan-HAA ggt gtt ttg ggg tca atc atc cat tac cct

(B) *Analysis of synonymous versus non-synonymous nucleotide substitution rates in teleost HAA cytoplasmic tail motif encoding fragments by* *SNAP v2.1.1 software*

The teleost *HAA* sequence fragments shown in (A) were analyzed by the Synonymous Non-synonymous Analysis Program, SNAP v2.1.1.

Sd: The number of observed synonymous substitutions; Sn: The number of observed non-synonymous substitutions; S: The number of potential synonymous substitutions (the average for the two compared sequences); N: The number of potential non-synonymous substitutions (the average for the two compared sequences); ps: The proportion of observed synonymous substitutions: Sd/S; pn: The proportion of observed non-synonymous substitutions: Sn/N; ds: The Jukes-Cantor correction for multiple hits of ps; dn: The Jukes-Cantor correction for multiple hits of pn

Summary of Results

*Averages of all pairwise comparisons:*

ds = 1.2992 dn = 0.1193 ds/dn = 14.6019 ps/pn = 6.5842

*Averages of the first sequence compared to others:*

ds = 1.2827 dn = 0.1655 ds/dn = 8.6887 ps/pn = 4.5030

*Statistics:*

Average ps: 0.686777; Variance (ps): 0.005329; Std Deviation(ps): 0.072997;

Average pn: 0.097732; Variance(pn): 0.001481; Std Deviation(pn): 0.038481;

Average dn: 0.108417

(B continued)

Detailed Results

Compare Sequence names Sd Sn S N ps pn ds dn ds/dn ps/pn

0 1 Orni-HAA GAAC-HAA 5.0000 2.0000 7.5000 22.5000 0.6667 0.0889 1.6479 0.0946 17.4174 7.5000

0 2 Orni-HAA Lacr-HAA 4.5000 3.5000 7.6667 22.3333 0.5870 0.1567 1.1445 0.1758 6.5105 3.7453

0 3 Orni-HAA Pore-HAA 4.0000 2.0000 7.5000 22.5000 0.5333 0.0889 0.9313 0.0946 9.8430 6.0000

0 4 Orni-HAA Teni-HAA 5.5000 3.5000 7.6667 22.3333 0.7174 0.1567 2.3516 0.1758 13.3766 4.5776

0 5 Orni-HAA Sasa-HAA 4.0000 2.0000 7.8333 22.1667 0.5106 0.0902 0.8566 0.0961 8.9104 5.6596

0 6 Orni-HAA Onmy-HAA 4.0000 2.0000 7.5000 22.5000 0.5333 0.0889 0.9313 0.0946 9.8430 6.0000

0 7 Orni-HAA Onki-HAA 4.0000 2.0000 7.5000 22.5000 0.5333 0.0889 0.9313 0.0946 9.8430 6.0000

0 8 Orni-HAA Onki-HBA 5.0000 2.0000 7.8333 22.1667 0.6383 0.0902 1.4282 0.0961 14.8565 7.0745

0 9 Orni-HAA Eslu-HAA 7.0000 2.0000 7.5000 22.5000 0.9333 0.0889 nan 0.0946 nan 10.5000

0 10 Orni-HAA Cyca-HAA 4.0000 4.0000 7.5000 22.5000 0.5333 0.1778 0.9313 0.2029 4.5897 3.0000

0 11 Orni-HAA Cyca-HAA 3.0000 4.0000 7.5000 22.5000 0.4000 0.1778 0.5716 0.2029 2.8170 2.2500

0 12 Orni-HAA Sirh-HAA 4.0000 4.0000 7.5000 22.5000 0.5333 0.1778 0.9313 0.2029 4.5897 3.0000

0 13 Orni-HAA Dare-HAA 5.0000 3.0000 7.6667 22.3333 0.6522 0.1343 1.5277 0.1480 10.3207 4.8551

0 14 Orni-HAA Asme-HAA 6.0000 4.0000 7.5000 22.5000 0.8000 0.1778 nan 0.2029 nan 4.5000

0 15 Orni-HAA Pime-HAA 6.5000 3.5000 7.5000 22.5000 0.8667 0.1556 nan 0.1743 nan 5.5714

0 16 Orni-HAA Coma-HAA 7.5000 3.5000 7.5000 22.5000 1.0000 0.1556 nan 0.1743 nan 6.4286

0 17 Orni-HAA Icpu-HAA 5.0000 5.0000 7.6667 22.3333 0.6522 0.2239 1.5277 0.2659 5.7451 2.9130

0 18 Orni-HAA Icfu-HAA 5.0000 5.0000 7.6667 22.3333 0.6522 0.2239 1.5277 0.2659 5.7451 2.9130

0 19 Orni-HAA Sime-HAA 7.5000 4.5000 7.6667 22.3333 0.9783 0.2015 nan 0.2347 nan 4.8551

0 20 Orni-HAA Sias-HAA 5.5000 4.5000 7.6667 22.3333 0.7174 0.2015 2.3516 0.2347 10.0216 3.5604

0 21 Orni-HAA Eivi-HAA 6.0000 3.0000 7.6667 22.3333 0.7826 0.1343 nan 0.1480 nan 5.8261

0 22 Orni-HAA Alal-HAA 6.0000 4.0000 7.6667 22.3333 0.7826 0.1791 nan 0.2047 nan 4.3696

0 23 Orni-HAA Clha-HAA 7.0000 3.0000 8.0000 22.0000 0.8750 0.1364 nan 0.1505 nan 6.4167

0 24 Orni-HAA Teil-HAA 8.0000 4.0000 7.8333 22.1667 1.0213 0.1805 nan 0.2064 nan 5.6596

0 25 Orni-HAA Sapi-HAA 6.0000 4.0000 8.0000 22.0000 0.7500 0.1818 nan 0.2082 nan 4.1250

0 26 Orni-HAA Anan-HAA 4.0000 4.0000 7.5000 22.5000 0.5333 0.1778 0.9313 0.2029 4.5897 3.0000

1 2 GAAC-HAA Lacr-HAA 5.0000 1.0000 7.8333 22.1667 0.6383 0.0451 1.4282 0.0465 30.6960 14.1489

1 3 GAAC-HAA Pore-HAA 7.0000 0.0000 7.6667 22.3333 0.9130 0.0000 nan 0.0000 nan nan

1 4 GAAC-HAA Teni-HAA 5.5000 1.5000 7.8333 22.1667 0.7021 0.0677 2.0637 0.0709 29.0987 10.3759

1 5 GAAC-HAA Sasa-HAA 5.0000 2.0000 8.0000 22.0000 0.6250 0.0909 1.3438 0.0969 13.8668 6.8750

1 6 GAAC-HAA Onmy-HAA 5.0000 4.0000 7.6667 22.3333 0.6522 0.1791 1.5277 0.2047 7.4647 3.6413

1 7 GAAC-HAA Onki-HAA 5.0000 4.0000 7.6667 22.3333 0.6522 0.1791 1.5277 0.2047 7.4647 3.6413

1 8 GAAC-HAA Onki-HBA 7.0000 2.0000 8.0000 22.0000 0.8750 0.0909 nan 0.0969 nan 9.6250

1 9 GAAC-HAA Eslu-HAA 8.0000 0.0000 7.6667 22.3333 1.0435 0.0000 nan 0.0000 nan nan

1 10 GAAC-HAA Cyca-HAA 5.5000 1.5000 7.6667 22.3333 0.7174 0.0672 2.3516 0.0704 33.4208 10.6812

1 11 GAAC-HAA Cyca-HAA 6.5000 1.5000 7.6667 22.3333 0.8478 0.0672 nan 0.0704 nan 12.6232

1 12 GAAC-HAA Sirh-HAA 5.5000 1.5000 7.6667 22.3333 0.7174 0.0672 2.3516 0.0704 33.4208 10.6812

1 13 GAAC-HAA Dare-HAA 5.5000 2.5000 7.8333 22.1667 0.7021 0.1128 2.0637 0.1222 16.8846 6.2255

1 14 GAAC-HAA Asme-HAA 6.0000 2.0000 7.6667 22.3333 0.7826 0.0896 nan 0.0954 nan 8.7391

1 15 GAAC-HAA Pime-HAA 6.5000 1.5000 7.6667 22.3333 0.8478 0.0672 nan 0.0704 nan 12.6232

1 16 GAAC-HAA Coma-HAA 6.5000 1.5000 7.6667 22.3333 0.8478 0.0672 nan 0.0704 nan 12.6232

1 17 GAAC-HAA Icpu-HAA 5.5000 2.5000 7.8333 22.1667 0.7021 0.1128 2.0637 0.1222 16.8846 6.2255

1 18 GAAC-HAA Icfu-HAA 5.5000 2.5000 7.8333 22.1667 0.7021 0.1128 2.0637 0.1222 16.8846 6.2255

1 19 GAAC-HAA Sime-HAA 6.5000 2.5000 7.8333 22.1667 0.8298 0.1128 nan 0.1222 nan 7.3574

1 20 GAAC-HAA Sias-HAA 4.5000 2.5000 7.8333 22.1667 0.5745 0.1128 1.0892 0.1222 8.9116 5.0936

1 21 GAAC-HAA Eivi-HAA 5.0000 1.0000 7.8333 22.1667 0.6383 0.0451 1.4282 0.0465 30.6960 14.1489

1 22 GAAC-HAA Alal-HAA 6.5000 3.5000 7.8333 22.1667 0.8298 0.1579 nan 0.1773 nan 5.2553

1 23 GAAC-HAA Clha-HAA 7.5000 2.5000 8.1667 21.8333 0.9184 0.1145 nan 0.1243 nan 8.0204

1 24 GAAC-HAA Teil-HAA 6.5000 1.5000 8.0000 22.0000 0.8125 0.0682 nan 0.0715 nan 11.9167

1 25 GAAC-HAA Sapi-HAA 6.3333 3.6667 8.1667 21.8333 0.7755 0.1679 nan 0.1901 nan 4.6178

1 26 GAAC-HAA Anan-HAA 6.0000 2.0000 7.6667 22.3333 0.7826 0.0896 nan 0.0954 nan 8.7391

2 3 Lacr-HAA Pore-HAA 6.0000 1.0000 7.8333 22.1667 0.7660 0.0451 nan 0.0465 nan 16.9787

2 4 Lacr-HAA Teni-HAA 6.0000 0.0000 8.0000 22.0000 0.7500 0.0000 nan 0.0000 nan nan

2 5 Lacr-HAA Sasa-HAA 5.0000 3.0000 8.1667 21.8333 0.6122 0.1374 1.2709 0.1518 8.3738 4.4558

2 6 Lacr-HAA Onmy-HAA 4.5000 5.5000 7.8333 22.1667 0.5745 0.2481 1.0892 0.3013 3.6152 2.3153

2 7 Lacr-HAA Onki-HAA 4.5000 5.5000 7.8333 22.1667 0.5745 0.2481 1.0892 0.3013 3.6152 2.3153

2 8 Lacr-HAA Onki-HBA 5.0000 3.0000 8.1667 21.8333 0.6122 0.1374 1.2709 0.1518 8.3738 4.4558

2 9 Lacr-HAA Eslu-HAA 7.0000 1.0000 7.8333 22.1667 0.8936 0.0451 nan 0.0465 nan 19.8085

2 10 Lacr-HAA Cyca-HAA 5.5000 2.5000 7.8333 22.1667 0.7021 0.1128 2.0637 0.1222 16.8846 6.2255

2 11 Lacr-HAA Cyca-HAA 4.5000 2.5000 7.8333 22.1667 0.5745 0.1128 1.0892 0.1222 8.9116 5.0936

2 12 Lacr-HAA Sirh-HAA 5.5000 2.5000 7.8333 22.1667 0.7021 0.1128 2.0637 0.1222 16.8846 6.2255

2 13 Lacr-HAA Dare-HAA 6.5000 3.5000 8.0000 22.0000 0.8125 0.1591 nan 0.1788 nan 5.1071

2 14 Lacr-HAA Asme-HAA 4.0000 3.0000 7.8333 22.1667 0.5106 0.1353 0.8566 0.1493 5.7391 3.7730

2 15 Lacr-HAA Pime-HAA 4.5000 2.5000 7.8333 22.1667 0.5745 0.1128 1.0892 0.1222 8.9116 5.0936

2 16 Lacr-HAA Coma-HAA 5.5000 2.5000 7.8333 22.1667 0.7021 0.1128 2.0637 0.1222 16.8846 6.2255

2 17 Lacr-HAA Icpu-HAA 4.5000 2.5000 8.0000 22.0000 0.5625 0.1136 1.0397 0.1232 8.4374 4.9500

2 18 Lacr-HAA Icfu-HAA 4.5000 2.5000 8.0000 22.0000 0.5625 0.1136 1.0397 0.1232 8.4374 4.9500

2 19 Lacr-HAA Sime-HAA 5.5000 2.5000 8.0000 22.0000 0.6875 0.1136 1.8637 0.1232 15.1239 6.0500

2 20 Lacr-HAA Sias-HAA 5.5000 2.5000 8.0000 22.0000 0.6875 0.1136 1.8637 0.1232 15.1239 6.0500

2 21 Lacr-HAA Eivi-HAA 4.0000 2.0000 8.0000 22.0000 0.5000 0.0909 0.8240 0.0969 8.5024 5.5000

2 22 Lacr-HAA Alal-HAA 6.5000 4.5000 8.0000 22.0000 0.8125 0.2045 nan 0.2388 nan 3.9722

2 23 Lacr-HAA Clha-HAA 7.5000 3.5000 8.3333 21.6667 0.9000 0.1615 nan 0.1819 nan 5.5714

2 24 Lacr-HAA Teil-HAA 7.5000 2.5000 8.1667 21.8333 0.9184 0.1145 nan 0.1243 nan 8.0204

2 25 Lacr-HAA Sapi-HAA 6.3333 4.6667 8.3333 21.6667 0.7600 0.2154 nan 0.2539 nan 3.5286

2 26 Lacr-HAA Anan-HAA 4.0000 3.0000 7.8333 22.1667 0.5106 0.1353 0.8566 0.1493 5.7391 3.7730

3 4 Pore-HAA Teni-HAA 7.5000 1.5000 7.8333 22.1667 0.9574 0.0677 nan 0.0709 nan 14.1489

3 5 Pore-HAA Sasa-HAA 5.0000 2.0000 8.0000 22.0000 0.6250 0.0909 1.3438 0.0969 13.8668 6.8750

3 6 Pore-HAA Onmy-HAA 5.0000 4.0000 7.6667 22.3333 0.6522 0.1791 1.5277 0.2047 7.4647 3.6413

3 7 Pore-HAA Onki-HAA 5.0000 4.0000 7.6667 22.3333 0.6522 0.1791 1.5277 0.2047 7.4647 3.6413

3 8 Pore-HAA Onki-HBA 7.0000 2.0000 8.0000 22.0000 0.8750 0.0909 nan 0.0969 nan 9.6250

3 9 Pore-HAA Eslu-HAA 7.0000 0.0000 7.6667 22.3333 0.9130 0.0000 nan 0.0000 nan nan

3 10 Pore-HAA Cyca-HAA 6.5000 1.5000 7.6667 22.3333 0.8478 0.0672 nan 0.0704 nan 12.6232

3 11 Pore-HAA Cyca-HAA 5.5000 1.5000 7.6667 22.3333 0.7174 0.0672 2.3516 0.0704 33.4208 10.6812

3 12 Pore-HAA Sirh-HAA 6.5000 1.5000 7.6667 22.3333 0.8478 0.0672 nan 0.0704 nan 12.6232

3 13 Pore-HAA Dare-HAA 7.5000 2.5000 7.8333 22.1667 0.9574 0.1128 nan 0.1222 nan 8.4894

3 14 Pore-HAA Asme-HAA 6.0000 2.0000 7.6667 22.3333 0.7826 0.0896 nan 0.0954 nan 8.7391

3 15 Pore-HAA Pime-HAA 6.5000 1.5000 7.6667 22.3333 0.8478 0.0672 nan 0.0704 nan 12.6232

3 16 Pore-HAA Coma-HAA 7.5000 1.5000 7.6667 22.3333 0.9783 0.0672 nan 0.0704 nan 14.5652

3 17 Pore-HAA Icpu-HAA 6.5000 2.5000 7.8333 22.1667 0.8298 0.1128 nan 0.1222 nan 7.3574

3 18 Pore-HAA Icfu-HAA 6.5000 2.5000 7.8333 22.1667 0.8298 0.1128 nan 0.1222 nan 7.3574

3 19 Pore-HAA Sime-HAA 7.5000 2.5000 7.8333 22.1667 0.9574 0.1128 nan 0.1222 nan 8.4894

3 20 Pore-HAA Sias-HAA 6.5000 2.5000 7.8333 22.1667 0.8298 0.1128 nan 0.1222 nan 7.3574

3 21 Pore-HAA Eivi-HAA 7.0000 1.0000 7.8333 22.1667 0.8936 0.0451 nan 0.0465 nan 19.8085

3 22 Pore-HAA Alal-HAA 8.0000 4.0000 7.8333 22.1667 1.0213 0.1805 nan 0.2064 nan 5.6596

3 23 Pore-HAA Clha-HAA 7.5000 2.5000 8.1667 21.8333 0.9184 0.1145 nan 0.1243 nan 8.0204

3 24 Pore-HAA Teil-HAA 8.5000 1.5000 8.0000 22.0000 1.0625 0.0682 nan 0.0715 nan 15.5833

3 25 Pore-HAA Sapi-HAA 8.5000 3.5000 8.1667 21.8333 1.0408 0.1603 nan 0.1804 nan 6.4927

3 26 Pore-HAA Anan-HAA 6.0000 2.0000 7.6667 22.3333 0.7826 0.0896 nan 0.0954 nan 8.7391

4 5 Teni-HAA Sasa-HAA 8.5000 3.5000 8.1667 21.8333 1.0408 0.1603 nan 0.1804 nan 6.4927

4 6 Teni-HAA Onmy-HAA 8.5000 5.5000 7.8333 22.1667 1.0851 0.2481 nan 0.3013 nan 4.3733

4 7 Teni-HAA Onki-HAA 8.5000 5.5000 7.8333 22.1667 1.0851 0.2481 nan 0.3013 nan 4.3733

4 8 Teni-HAA Onki-HBA 7.5000 3.5000 8.1667 21.8333 0.9184 0.1603 nan 0.1804 nan 5.7289

4 9 Teni-HAA Eslu-HAA 7.5000 1.5000 7.8333 22.1667 0.9574 0.0677 nan 0.0709 nan 14.1489

4 10 Teni-HAA Cyca-HAA 6.0000 3.0000 7.8333 22.1667 0.7660 0.1353 nan 0.1493 nan 5.6596

4 11 Teni-HAA Cyca-HAA 5.0000 3.0000 7.8333 22.1667 0.6383 0.1353 1.4282 0.1493 9.5690 4.7163

4 12 Teni-HAA Sirh-HAA 6.0000 3.0000 7.8333 22.1667 0.7660 0.1353 nan 0.1493 nan 5.6596

4 13 Teni-HAA Dare-HAA 6.0000 4.0000 8.0000 22.0000 0.7500 0.1818 nan 0.2082 nan 4.1250

4 14 Teni-HAA Asme-HAA 6.5000 3.5000 7.8333 22.1667 0.8298 0.1579 nan 0.1773 nan 5.2553

4 15 Teni-HAA Pime-HAA 7.0000 3.0000 7.8333 22.1667 0.8936 0.1353 nan 0.1493 nan 6.6028

4 16 Teni-HAA Coma-HAA 6.0000 3.0000 7.8333 22.1667 0.7660 0.1353 nan 0.1493 nan 5.6596

4 17 Teni-HAA Icpu-HAA 6.5000 2.5000 8.0000 22.0000 0.8125 0.1136 nan 0.1232 nan 7.1500

4 18 Teni-HAA Icfu-HAA 6.5000 2.5000 8.0000 22.0000 0.8125 0.1136 nan 0.1232 nan 7.1500

4 19 Teni-HAA Sime-HAA 6.5000 2.5000 8.0000 22.0000 0.8125 0.1136 nan 0.1232 nan 7.1500

4 20 Teni-HAA Sias-HAA 6.5000 2.5000 8.0000 22.0000 0.8125 0.1136 nan 0.1232 nan 7.1500

4 21 Teni-HAA Eivi-HAA 6.5000 2.5000 8.0000 22.0000 0.8125 0.1136 nan 0.1232 nan 7.1500

4 22 Teni-HAA Alal-HAA 5.0000 5.0000 8.0000 22.0000 0.6250 0.2273 1.3438 0.2708 4.9631 2.7500

4 23 Teni-HAA Clha-HAA 5.0000 4.0000 8.3333 21.6667 0.6000 0.1846 1.2071 0.2119 5.6958 3.2500

4 24 Teni-HAA Teil-HAA 6.0000 3.0000 8.1667 21.8333 0.7347 0.1374 2.9189 0.1518 19.2314 5.3469

4 25 Teni-HAA Sapi-HAA 4.8333 5.1667 8.3333 21.6667 0.5800 0.2385 1.1132 0.2870 3.8789 2.4323

4 26 Teni-HAA Anan-HAA 5.5000 3.5000 7.8333 22.1667 0.7021 0.1579 2.0637 0.1773 11.6399 4.4468

5 6 Sasa-HAA Onmy-HAA 0.0000 2.0000 8.0000 22.0000 0.0000 0.0909 0.0000 0.0969 nan nan

5 7 Sasa-HAA Onki-HAA 0.0000 2.0000 8.0000 22.0000 0.0000 0.0909 0.0000 0.0969 nan nan

5 8 Sasa-HAA Onki-HBA 3.0000 0.0000 8.3333 21.6667 0.3600 0.0000 0.4904 0.0000 nan nan

5 9 Sasa-HAA Eslu-HAA 5.0000 2.0000 8.0000 22.0000 0.6250 0.0909 1.3438 0.0969 13.8668 6.8750

5 10 Sasa-HAA Cyca-HAA 5.0000 2.0000 8.0000 22.0000 0.6250 0.0909 1.3438 0.0969 13.8668 6.8750

5 11 Sasa-HAA Cyca-HAA 6.0000 2.0000 8.0000 22.0000 0.7500 0.0909 nan 0.0969 nan 8.2500

5 12 Sasa-HAA Sirh-HAA 5.0000 2.0000 8.0000 22.0000 0.6250 0.0909 1.3438 0.0969 13.8668 6.8750

5 13 Sasa-HAA Dare-HAA 6.0000 1.0000 8.1667 21.8333 0.7347 0.0458 2.9189 0.0473 61.7622 16.0408

5 14 Sasa-HAA Asme-HAA 4.0000 2.0000 8.0000 22.0000 0.5000 0.0909 0.8240 0.0969 8.5024 5.5000

5 15 Sasa-HAA Pime-HAA 4.0000 2.0000 8.0000 22.0000 0.5000 0.0909 0.8240 0.0969 8.5024 5.5000

5 16 Sasa-HAA Coma-HAA 5.0000 2.0000 8.0000 22.0000 0.6250 0.0909 1.3438 0.0969 13.8668 6.8750

5 17 Sasa-HAA Icpu-HAA 7.0000 3.0000 8.1667 21.8333 0.8571 0.1374 nan 0.1518 nan 6.2381

5 18 Sasa-HAA Icfu-HAA 7.0000 3.0000 8.1667 21.8333 0.8571 0.1374 nan 0.1518 nan 6.2381

5 19 Sasa-HAA Sime-HAA 8.0000 3.0000 8.1667 21.8333 0.9796 0.1374 nan 0.1518 nan 7.1293

5 20 Sasa-HAA Sias-HAA 6.0000 3.0000 8.1667 21.8333 0.7347 0.1374 2.9189 0.1518 19.2314 5.3469

5 21 Sasa-HAA Eivi-HAA 3.0000 1.0000 8.1667 21.8333 0.3673 0.0458 0.5047 0.0473 10.6795 8.0204

5 22 Sasa-HAA Alal-HAA 7.0000 2.0000 8.1667 21.8333 0.8571 0.0916 nan 0.0977 nan 9.3571

5 23 Sasa-HAA Clha-HAA 8.0000 0.0000 8.5000 21.5000 0.9412 0.0000 nan 0.0000 nan nan

5 24 Sasa-HAA Teil-HAA 8.0000 1.0000 8.3333 21.6667 0.9600 0.0462 nan 0.0476 nan 20.8000

5 25 Sasa-HAA Sapi-HAA 7.0000 1.0000 8.5000 21.5000 0.8235 0.0465 nan 0.0480 nan 17.7059

5 26 Sasa-HAA Anan-HAA 6.0000 2.0000 8.0000 22.0000 0.7500 0.0909 nan 0.0969 nan 8.2500

6 7 Onmy-HAA Onki-HAA 0.0000 0.0000 7.6667 22.3333 0.0000 0.0000 0.0000 0.0000 nan nan

6 8 Onmy-HAA Onki-HBA 3.0000 2.0000 8.0000 22.0000 0.3750 0.0909 0.5199 0.0969 5.3644 4.1250

6 9 Onmy-HAA Eslu-HAA 5.0000 4.0000 7.6667 22.3333 0.6522 0.1791 1.5277 0.2047 7.4647 3.6413

6 10 Onmy-HAA Cyca-HAA 4.5000 4.5000 7.6667 22.3333 0.5870 0.2015 1.1445 0.2347 4.8776 2.9130

6 11 Onmy-HAA Cyca-HAA 5.5000 4.5000 7.6667 22.3333 0.7174 0.2015 2.3516 0.2347 10.0216 3.5604

6 12 Onmy-HAA Sirh-HAA 4.5000 4.5000 7.6667 22.3333 0.5870 0.2015 1.1445 0.2347 4.8776 2.9130

6 13 Onmy-HAA Dare-HAA 5.5000 3.5000 7.8333 22.1667 0.7021 0.1579 2.0637 0.1773 11.6399 4.4468

6 14 Onmy-HAA Asme-HAA 4.0000 4.0000 7.6667 22.3333 0.5217 0.1791 0.8922 0.2047 4.3596 2.9130

6 15 Onmy-HAA Pime-HAA 4.0000 4.0000 7.6667 22.3333 0.5217 0.1791 0.8922 0.2047 4.3596 2.9130

6 16 Onmy-HAA Coma-HAA 5.0000 4.0000 7.6667 22.3333 0.6522 0.1791 1.5277 0.2047 7.4647 3.6413

6 17 Onmy-HAA Icpu-HAA 6.5000 5.5000 7.8333 22.1667 0.8298 0.2481 nan 0.3013 nan 3.3443

6 18 Onmy-HAA Icfu-HAA 6.5000 5.5000 7.8333 22.1667 0.8298 0.2481 nan 0.3013 nan 3.3443

6 19 Onmy-HAA Sime-HAA 8.0000 5.0000 7.8333 22.1667 1.0213 0.2256 nan 0.2683 nan 4.5277

6 20 Onmy-HAA Sias-HAA 6.0000 5.0000 7.8333 22.1667 0.7660 0.2256 nan 0.2683 nan 3.3957

6 21 Onmy-HAA Eivi-HAA 3.0000 3.0000 7.8333 22.1667 0.3830 0.1353 0.5360 0.1493 3.5912 2.8298

6 22 Onmy-HAA Alal-HAA 6.5000 4.5000 7.8333 22.1667 0.8298 0.2030 nan 0.2367 nan 4.0875

6 23 Onmy-HAA Clha-HAA 7.5000 2.5000 8.1667 21.8333 0.9184 0.1145 nan 0.1243 nan 8.0204

6 24 Onmy-HAA Teil-HAA 7.5000 3.5000 8.0000 22.0000 0.9375 0.1591 nan 0.1788 nan 5.8929

6 25 Onmy-HAA Sapi-HAA 6.5000 3.5000 8.1667 21.8333 0.7959 0.1603 nan 0.1804 nan 4.9650

6 26 Onmy-HAA Anan-HAA 6.0000 4.0000 7.6667 22.3333 0.7826 0.1791 nan 0.2047 nan 4.3696

7 8 Onki-HAA Onki-HBA 3.0000 2.0000 8.0000 22.0000 0.3750 0.0909 0.5199 0.0969 5.3644 4.1250

7 9 Onki-HAA Eslu-HAA 5.0000 4.0000 7.6667 22.3333 0.6522 0.1791 1.5277 0.2047 7.4647 3.6413

7 10 Onki-HAA Cyca-HAA 4.5000 4.5000 7.6667 22.3333 0.5870 0.2015 1.1445 0.2347 4.8776 2.9130

7 11 Onki-HAA Cyca-HAA 5.5000 4.5000 7.6667 22.3333 0.7174 0.2015 2.3516 0.2347 10.0216 3.5604

7 12 Onki-HAA Sirh-HAA 4.5000 4.5000 7.6667 22.3333 0.5870 0.2015 1.1445 0.2347 4.8776 2.9130

7 13 Onki-HAA Dare-HAA 5.5000 3.5000 7.8333 22.1667 0.7021 0.1579 2.0637 0.1773 11.6399 4.4468

7 14 Onki-HAA Asme-HAA 4.0000 4.0000 7.6667 22.3333 0.5217 0.1791 0.8922 0.2047 4.3596 2.9130

7 15 Onki-HAA Pime-HAA 4.0000 4.0000 7.6667 22.3333 0.5217 0.1791 0.8922 0.2047 4.3596 2.9130

7 16 Onki-HAA Coma-HAA 5.0000 4.0000 7.6667 22.3333 0.6522 0.1791 1.5277 0.2047 7.4647 3.6413

7 17 Onki-HAA Icpu-HAA 6.5000 5.5000 7.8333 22.1667 0.8298 0.2481 nan 0.3013 nan 3.3443

7 18 Onki-HAA Icfu-HAA 6.5000 5.5000 7.8333 22.1667 0.8298 0.2481 nan 0.3013 nan 3.3443

7 19 Onki-HAA Sime-HAA 8.0000 5.0000 7.8333 22.1667 1.0213 0.2256 nan 0.2683 nan 4.5277

7 20 Onki-HAA Sias-HAA 6.0000 5.0000 7.8333 22.1667 0.7660 0.2256 nan 0.2683 nan 3.3957

7 21 Onki-HAA Eivi-HAA 3.0000 3.0000 7.8333 22.1667 0.3830 0.1353 0.5360 0.1493 3.5912 2.8298

7 22 Onki-HAA Alal-HAA 6.5000 4.5000 7.8333 22.1667 0.8298 0.2030 nan 0.2367 nan 4.0875

7 23 Onki-HAA Clha-HAA 7.5000 2.5000 8.1667 21.8333 0.9184 0.1145 nan 0.1243 nan 8.0204

7 24 Onki-HAA Teil-HAA 7.5000 3.5000 8.0000 22.0000 0.9375 0.1591 nan 0.1788 nan 5.8929

7 25 Onki-HAA Sapi-HAA 6.5000 3.5000 8.1667 21.8333 0.7959 0.1603 nan 0.1804 nan 4.9650

7 26 Onki-HAA Anan-HAA 6.0000 4.0000 7.6667 22.3333 0.7826 0.1791 nan 0.2047 nan 4.3696

8 9 Onki-HBA Eslu-HAA 3.0000 2.0000 8.0000 22.0000 0.3750 0.0909 0.5199 0.0969 5.3644 4.1250

8 10 Onki-HBA Cyca-HAA 5.0000 2.0000 8.0000 22.0000 0.6250 0.0909 1.3438 0.0969 13.8668 6.8750

8 11 Onki-HBA Cyca-HAA 6.0000 2.0000 8.0000 22.0000 0.7500 0.0909 nan 0.0969 nan 8.2500

8 12 Onki-HBA Sirh-HAA 5.0000 2.0000 8.0000 22.0000 0.6250 0.0909 1.3438 0.0969 13.8668 6.8750

8 13 Onki-HBA Dare-HAA 6.0000 1.0000 8.1667 21.8333 0.7347 0.0458 2.9189 0.0473 61.7622 16.0408

8 14 Onki-HBA Asme-HAA 2.0000 2.0000 8.0000 22.0000 0.2500 0.0909 0.3041 0.0969 3.1380 2.7500

8 15 Onki-HBA Pime-HAA 2.0000 2.0000 8.0000 22.0000 0.2500 0.0909 0.3041 0.0969 3.1380 2.7500

8 16 Onki-HBA Coma-HAA 3.0000 2.0000 8.0000 22.0000 0.3750 0.0909 0.5199 0.0969 5.3644 4.1250

8 17 Onki-HBA Icpu-HAA 7.0000 3.0000 8.1667 21.8333 0.8571 0.1374 nan 0.1518 nan 6.2381

8 18 Onki-HBA Icfu-HAA 7.0000 3.0000 8.1667 21.8333 0.8571 0.1374 nan 0.1518 nan 6.2381

8 19 Onki-HBA Sime-HAA 8.0000 3.0000 8.1667 21.8333 0.9796 0.1374 nan 0.1518 nan 7.1293

8 20 Onki-HBA Sias-HAA 8.0000 3.0000 8.1667 21.8333 0.9796 0.1374 nan 0.1518 nan 7.1293

8 21 Onki-HBA Eivi-HAA 3.0000 1.0000 8.1667 21.8333 0.3673 0.0458 0.5047 0.0473 10.6795 8.0204

8 22 Onki-HBA Alal-HAA 6.0000 2.0000 8.1667 21.8333 0.7347 0.0916 2.9189 0.0977 29.8761 8.0204

8 23 Onki-HBA Clha-HAA 7.0000 0.0000 8.5000 21.5000 0.8235 0.0000 nan 0.0000 nan nan

8 24 Onki-HBA Teil-HAA 7.0000 1.0000 8.3333 21.6667 0.8400 0.0462 nan 0.0476 nan 18.2000

8 25 Onki-HBA Sapi-HAA 6.0000 1.0000 8.5000 21.5000 0.7059 0.0465 2.1249 0.0480 44.2538 15.1765

8 26 Onki-HBA Anan-HAA 6.0000 2.0000 8.0000 22.0000 0.7500 0.0909 nan 0.0969 nan 8.2500

9 10 Eslu-HAA Cyca-HAA 6.5000 1.5000 7.6667 22.3333 0.8478 0.0672 nan 0.0704 nan 12.6232

9 11 Eslu-HAA Cyca-HAA 7.5000 1.5000 7.6667 22.3333 0.9783 0.0672 nan 0.0704 nan 14.5652

9 12 Eslu-HAA Sirh-HAA 6.5000 1.5000 7.6667 22.3333 0.8478 0.0672 nan 0.0704 nan 12.6232

9 13 Eslu-HAA Dare-HAA 7.5000 2.5000 7.8333 22.1667 0.9574 0.1128 nan 0.1222 nan 8.4894

9 14 Eslu-HAA Asme-HAA 4.0000 2.0000 7.6667 22.3333 0.5217 0.0896 0.8922 0.0954 9.3554 5.8261

9 15 Eslu-HAA Pime-HAA 4.5000 1.5000 7.6667 22.3333 0.5870 0.0672 1.1445 0.0704 16.2660 8.7391

9 16 Eslu-HAA Coma-HAA 5.5000 1.5000 7.6667 22.3333 0.7174 0.0672 2.3516 0.0704 33.4208 10.6812

9 17 Eslu-HAA Icpu-HAA 6.5000 2.5000 7.8333 22.1667 0.8298 0.1128 nan 0.1222 nan 7.3574

9 18 Eslu-HAA Icfu-HAA 6.5000 2.5000 7.8333 22.1667 0.8298 0.1128 nan 0.1222 nan 7.3574

9 19 Eslu-HAA Sime-HAA 7.5000 2.5000 7.8333 22.1667 0.9574 0.1128 nan 0.1222 nan 8.4894

9 20 Eslu-HAA Sias-HAA 7.5000 2.5000 7.8333 22.1667 0.9574 0.1128 nan 0.1222 nan 8.4894

9 21 Eslu-HAA Eivi-HAA 6.0000 1.0000 7.8333 22.1667 0.7660 0.0451 nan 0.0465 nan 16.9787

9 22 Eslu-HAA Alal-HAA 6.5000 3.5000 7.8333 22.1667 0.8298 0.1579 nan 0.1773 nan 5.2553

9 23 Eslu-HAA Clha-HAA 5.5000 2.5000 8.1667 21.8333 0.6735 0.1145 1.7118 0.1243 13.7769 5.8816

9 24 Eslu-HAA Teil-HAA 6.5000 1.5000 8.0000 22.0000 0.8125 0.0682 nan 0.0715 nan 11.9167

9 25 Eslu-HAA Sapi-HAA 6.5000 3.5000 8.1667 21.8333 0.7959 0.1603 nan 0.1804 nan 4.9650

9 26 Eslu-HAA Anan-HAA 8.0000 2.0000 7.6667 22.3333 1.0435 0.0896 nan 0.0954 nan 11.6522

10 11 Cyca-HAA Cyca-HAA 1.0000 0.0000 7.6667 22.3333 0.1304 0.0000 0.1433 0.0000 nan nan

10 12 Cyca-HAA Sirh-HAA 0.0000 0.0000 7.6667 22.3333 0.0000 0.0000 0.0000 0.0000 nan nan

10 13 Cyca-HAA Dare-HAA 1.0000 1.0000 7.8333 22.1667 0.1277 0.0451 0.1399 0.0465 3.0077 2.8298

10 14 Cyca-HAA Asme-HAA 4.0000 0.0000 7.6667 22.3333 0.5217 0.0000 0.8922 0.0000 nan nan

10 15 Cyca-HAA Pime-HAA 3.0000 0.0000 7.6667 22.3333 0.3913 0.0000 0.5532 0.0000 nan nan

10 16 Cyca-HAA Coma-HAA 4.0000 0.0000 7.6667 22.3333 0.5217 0.0000 0.8922 0.0000 nan nan

10 17 Cyca-HAA Icpu-HAA 3.0000 1.0000 7.8333 22.1667 0.3830 0.0451 0.5360 0.0465 11.5201 8.4894

10 18 Cyca-HAA Icfu-HAA 3.0000 1.0000 7.8333 22.1667 0.3830 0.0451 0.5360 0.0465 11.5201 8.4894

10 19 Cyca-HAA Sime-HAA 8.0000 1.0000 7.8333 22.1667 1.0213 0.0451 nan 0.0465 nan 22.6383

10 20 Cyca-HAA Sias-HAA 6.0000 1.0000 7.8333 22.1667 0.7660 0.0451 nan 0.0465 nan 16.9787

10 21 Cyca-HAA Eivi-HAA 6.0000 1.0000 7.8333 22.1667 0.7660 0.0451 nan 0.0465 nan 16.9787

10 22 Cyca-HAA Alal-HAA 3.0000 3.0000 7.8333 22.1667 0.3830 0.1353 0.5360 0.1493 3.5912 2.8298

10 23 Cyca-HAA Clha-HAA 5.0000 1.0000 8.1667 21.8333 0.6122 0.0458 1.2709 0.0473 26.8928 13.3673

10 24 Cyca-HAA Teil-HAA 6.0000 0.0000 8.0000 22.0000 0.7500 0.0000 nan 0.0000 nan nan

10 25 Cyca-HAA Sapi-HAA 4.0000 2.0000 8.1667 21.8333 0.4898 0.0916 0.7940 0.0977 8.1266 5.3469

10 26 Cyca-HAA Anan-HAA 6.0000 0.0000 7.6667 22.3333 0.7826 0.0000 nan 0.0000 nan nan

11 12 Cyca-HAA Sirh-HAA 1.0000 0.0000 7.6667 22.3333 0.1304 0.0000 0.1433 0.0000 nan nan

11 13 Cyca-HAA Dare-HAA 2.0000 1.0000 7.8333 22.1667 0.2553 0.0451 0.3121 0.0465 6.7084 5.6596

11 14 Cyca-HAA Asme-HAA 5.0000 0.0000 7.6667 22.3333 0.6522 0.0000 1.5277 0.0000 nan nan

11 15 Cyca-HAA Pime-HAA 4.0000 0.0000 7.6667 22.3333 0.5217 0.0000 0.8922 0.0000 nan nan

11 16 Cyca-HAA Coma-HAA 5.0000 0.0000 7.6667 22.3333 0.6522 0.0000 1.5277 0.0000 nan nan

11 17 Cyca-HAA Icpu-HAA 2.0000 1.0000 7.8333 22.1667 0.2553 0.0451 0.3121 0.0465 6.7084 5.6596

11 18 Cyca-HAA Icfu-HAA 2.0000 1.0000 7.8333 22.1667 0.2553 0.0451 0.3121 0.0465 6.7084 5.6596

11 19 Cyca-HAA Sime-HAA 7.0000 1.0000 7.8333 22.1667 0.8936 0.0451 nan 0.0465 nan 19.8085

11 20 Cyca-HAA Sias-HAA 5.0000 1.0000 7.8333 22.1667 0.6383 0.0451 1.4282 0.0465 30.6960 14.1489

11 21 Cyca-HAA Eivi-HAA 7.0000 1.0000 7.8333 22.1667 0.8936 0.0451 nan 0.0465 nan 19.8085

11 22 Cyca-HAA Alal-HAA 4.0000 3.0000 7.8333 22.1667 0.5106 0.1353 0.8566 0.1493 5.7391 3.7730

11 23 Cyca-HAA Clha-HAA 6.0000 1.0000 8.1667 21.8333 0.7347 0.0458 2.9189 0.0473 61.7622 16.0408

11 24 Cyca-HAA Teil-HAA 7.0000 0.0000 8.0000 22.0000 0.8750 0.0000 nan 0.0000 nan nan

11 25 Cyca-HAA Sapi-HAA 5.0000 2.0000 8.1667 21.8333 0.6122 0.0916 1.2709 0.0977 13.0088 6.6837

11 26 Cyca-HAA Anan-HAA 5.0000 0.0000 7.6667 22.3333 0.6522 0.0000 1.5277 0.0000 nan nan

12 13 Sirh-HAA Dare-HAA 1.0000 1.0000 7.8333 22.1667 0.1277 0.0451 0.1399 0.0465 3.0077 2.8298

12 14 Sirh-HAA Asme-HAA 4.0000 0.0000 7.6667 22.3333 0.5217 0.0000 0.8922 0.0000 nan nan

12 15 Sirh-HAA Pime-HAA 3.0000 0.0000 7.6667 22.3333 0.3913 0.0000 0.5532 0.0000 nan nan

12 16 Sirh-HAA Coma-HAA 4.0000 0.0000 7.6667 22.3333 0.5217 0.0000 0.8922 0.0000 nan nan

12 17 Sirh-HAA Icpu-HAA 3.0000 1.0000 7.8333 22.1667 0.3830 0.0451 0.5360 0.0465 11.5201 8.4894

12 18 Sirh-HAA Icfu-HAA 3.0000 1.0000 7.8333 22.1667 0.3830 0.0451 0.5360 0.0465 11.5201 8.4894

12 19 Sirh-HAA Sime-HAA 8.0000 1.0000 7.8333 22.1667 1.0213 0.0451 nan 0.0465 nan 22.6383

12 20 Sirh-HAA Sias-HAA 6.0000 1.0000 7.8333 22.1667 0.7660 0.0451 nan 0.0465 nan 16.9787

12 21 Sirh-HAA Eivi-HAA 6.0000 1.0000 7.8333 22.1667 0.7660 0.0451 nan 0.0465 nan 16.9787

12 22 Sirh-HAA Alal-HAA 3.0000 3.0000 7.8333 22.1667 0.3830 0.1353 0.5360 0.1493 3.5912 2.8298

12 23 Sirh-HAA Clha-HAA 5.0000 1.0000 8.1667 21.8333 0.6122 0.0458 1.2709 0.0473 26.8928 13.3673

12 24 Sirh-HAA Teil-HAA 6.0000 0.0000 8.0000 22.0000 0.7500 0.0000 nan 0.0000 nan nan

12 25 Sirh-HAA Sapi-HAA 4.0000 2.0000 8.1667 21.8333 0.4898 0.0916 0.7940 0.0977 8.1266 5.3469

12 26 Sirh-HAA Anan-HAA 6.0000 0.0000 7.6667 22.3333 0.7826 0.0000 nan 0.0000 nan nan

13 14 Dare-HAA Asme-HAA 5.0000 1.0000 7.8333 22.1667 0.6383 0.0451 1.4282 0.0465 30.6960 14.1489

13 15 Dare-HAA Pime-HAA 4.0000 1.0000 7.8333 22.1667 0.5106 0.0451 0.8566 0.0465 18.4104 11.3191

13 16 Dare-HAA Coma-HAA 4.0000 1.0000 7.8333 22.1667 0.5106 0.0451 0.8566 0.0465 18.4104 11.3191

13 17 Dare-HAA Icpu-HAA 4.0000 2.0000 8.0000 22.0000 0.5000 0.0909 0.8240 0.0969 8.5024 5.5000

13 18 Dare-HAA Icfu-HAA 4.0000 2.0000 8.0000 22.0000 0.5000 0.0909 0.8240 0.0969 8.5024 5.5000

13 19 Dare-HAA Sime-HAA 8.0000 2.0000 8.0000 22.0000 1.0000 0.0909 nan 0.0969 nan 11.0000

13 20 Dare-HAA Sias-HAA 6.0000 2.0000 8.0000 22.0000 0.7500 0.0909 nan 0.0969 nan 8.2500

13 21 Dare-HAA Eivi-HAA 6.0000 2.0000 8.0000 22.0000 0.7500 0.0909 nan 0.0969 nan 8.2500

13 22 Dare-HAA Alal-HAA 3.0000 2.0000 8.0000 22.0000 0.3750 0.0909 0.5199 0.0969 5.3644 4.1250

13 23 Dare-HAA Clha-HAA 5.0000 0.0000 8.3333 21.6667 0.6000 0.0000 1.2071 0.0000 nan nan

13 24 Dare-HAA Teil-HAA 6.0000 1.0000 8.1667 21.8333 0.7347 0.0458 2.9189 0.0473 61.7622 16.0408

13 25 Dare-HAA Sapi-HAA 4.0000 1.0000 8.3333 21.6667 0.4800 0.0462 0.7662 0.0476 16.0856 10.4000

13 26 Dare-HAA Anan-HAA 5.0000 1.0000 7.8333 22.1667 0.6383 0.0451 1.4282 0.0465 30.6960 14.1489

14 15 Asme-HAA Pime-HAA 1.0000 0.0000 7.6667 22.3333 0.1304 0.0000 0.1433 0.0000 nan nan

14 16 Asme-HAA Coma-HAA 2.0000 0.0000 7.6667 22.3333 0.2609 0.0000 0.3206 0.0000 nan nan

14 17 Asme-HAA Icpu-HAA 6.0000 1.0000 7.8333 22.1667 0.7660 0.0451 nan 0.0465 nan 16.9787

14 18 Asme-HAA Icfu-HAA 6.0000 1.0000 7.8333 22.1667 0.7660 0.0451 nan 0.0465 nan 16.9787

14 19 Asme-HAA Sime-HAA 7.0000 1.0000 7.8333 22.1667 0.8936 0.0451 nan 0.0465 nan 19.8085

14 20 Asme-HAA Sias-HAA 7.0000 1.0000 7.8333 22.1667 0.8936 0.0451 nan 0.0465 nan 19.8085

14 21 Asme-HAA Eivi-HAA 3.0000 1.0000 7.8333 22.1667 0.3830 0.0451 0.5360 0.0465 11.5201 8.4894

14 22 Asme-HAA Alal-HAA 4.0000 3.0000 7.8333 22.1667 0.5106 0.1353 0.8566 0.1493 5.7391 3.7730

14 23 Asme-HAA Clha-HAA 6.0000 1.0000 8.1667 21.8333 0.7347 0.0458 2.9189 0.0473 61.7622 16.0408

14 24 Asme-HAA Teil-HAA 6.0000 0.0000 8.0000 22.0000 0.7500 0.0000 nan 0.0000 nan nan

14 25 Asme-HAA Sapi-HAA 5.0000 2.0000 8.1667 21.8333 0.6122 0.0916 1.2709 0.0977 13.0088 6.6837

14 26 Asme-HAA Anan-HAA 4.0000 0.0000 7.6667 22.3333 0.5217 0.0000 0.8922 0.0000 nan nan

15 16 Pime-HAA Coma-HAA 1.0000 0.0000 7.6667 22.3333 0.1304 0.0000 0.1433 0.0000 nan nan

15 17 Pime-HAA Icpu-HAA 5.0000 1.0000 7.8333 22.1667 0.6383 0.0451 1.4282 0.0465 30.6960 14.1489

15 18 Pime-HAA Icfu-HAA 5.0000 1.0000 7.8333 22.1667 0.6383 0.0451 1.4282 0.0465 30.6960 14.1489

15 19 Pime-HAA Sime-HAA 6.0000 1.0000 7.8333 22.1667 0.7660 0.0451 nan 0.0465 nan 16.9787

15 20 Pime-HAA Sias-HAA 6.0000 1.0000 7.8333 22.1667 0.7660 0.0451 nan 0.0465 nan 16.9787

15 21 Pime-HAA Eivi-HAA 3.0000 1.0000 7.8333 22.1667 0.3830 0.0451 0.5360 0.0465 11.5201 8.4894

15 22 Pime-HAA Alal-HAA 5.0000 3.0000 7.8333 22.1667 0.6383 0.1353 1.4282 0.1493 9.5690 4.7163

15 23 Pime-HAA Clha-HAA 7.0000 1.0000 8.1667 21.8333 0.8571 0.0458 nan 0.0473 nan 18.7143

15 24 Pime-HAA Teil-HAA 7.0000 0.0000 8.0000 22.0000 0.8750 0.0000 nan 0.0000 nan nan

15 25 Pime-HAA Sapi-HAA 6.0000 2.0000 8.1667 21.8333 0.7347 0.0916 2.9189 0.0977 29.8761 8.0204

15 26 Pime-HAA Anan-HAA 5.0000 0.0000 7.6667 22.3333 0.6522 0.0000 1.5277 0.0000 nan nan

16 17 Coma-HAA Icpu-HAA 6.0000 1.0000 7.8333 22.1667 0.7660 0.0451 nan 0.0465 nan 16.9787

16 18 Coma-HAA Icfu-HAA 6.0000 1.0000 7.8333 22.1667 0.7660 0.0451 nan 0.0465 nan 16.9787

16 19 Coma-HAA Sime-HAA 5.0000 1.0000 7.8333 22.1667 0.6383 0.0451 1.4282 0.0465 30.6960 14.1489

16 20 Coma-HAA Sias-HAA 5.0000 1.0000 7.8333 22.1667 0.6383 0.0451 1.4282 0.0465 30.6960 14.1489

16 21 Coma-HAA Eivi-HAA 2.0000 1.0000 7.8333 22.1667 0.2553 0.0451 0.3121 0.0465 6.7084 5.6596

16 22 Coma-HAA Alal-HAA 4.0000 3.0000 7.8333 22.1667 0.5106 0.1353 0.8566 0.1493 5.7391 3.7730

16 23 Coma-HAA Clha-HAA 6.0000 1.0000 8.1667 21.8333 0.7347 0.0458 2.9189 0.0473 61.7622 16.0408

16 24 Coma-HAA Teil-HAA 6.0000 0.0000 8.0000 22.0000 0.7500 0.0000 nan 0.0000 nan nan

16 25 Coma-HAA Sapi-HAA 5.0000 2.0000 8.1667 21.8333 0.6122 0.0916 1.2709 0.0977 13.0088 6.6837

16 26 Coma-HAA Anan-HAA 5.0000 0.0000 7.6667 22.3333 0.6522 0.0000 1.5277 0.0000 nan nan

17 18 Icpu-HAA Icfu-HAA 0.0000 0.0000 8.0000 22.0000 0.0000 0.0000 0.0000 0.0000 nan nan

17 19 Icpu-HAA Sime-HAA 5.0000 0.0000 8.0000 22.0000 0.6250 0.0000 1.3438 0.0000 nan nan

17 20 Icpu-HAA Sias-HAA 3.0000 0.0000 8.0000 22.0000 0.3750 0.0000 0.5199 0.0000 nan nan

17 21 Icpu-HAA Eivi-HAA 8.0000 2.0000 8.0000 22.0000 1.0000 0.0909 nan 0.0969 nan 11.0000

17 22 Icpu-HAA Alal-HAA 6.0000 4.0000 8.0000 22.0000 0.7500 0.1818 nan 0.2082 nan 4.1250

17 23 Icpu-HAA Clha-HAA 8.0000 2.0000 8.3333 21.6667 0.9600 0.0923 nan 0.0985 nan 10.4000

17 24 Icpu-HAA Teil-HAA 7.0000 1.0000 8.1667 21.8333 0.8571 0.0458 nan 0.0473 nan 18.7143

17 25 Icpu-HAA Sapi-HAA 7.0000 3.0000 8.3333 21.6667 0.8400 0.1385 nan 0.1531 nan 6.0667

17 26 Icpu-HAA Anan-HAA 7.0000 1.0000 7.8333 22.1667 0.8936 0.0451 nan 0.0465 nan 19.8085

18 19 Icfu-HAA Sime-HAA 5.0000 0.0000 8.0000 22.0000 0.6250 0.0000 1.3438 0.0000 nan nan

18 20 Icfu-HAA Sias-HAA 3.0000 0.0000 8.0000 22.0000 0.3750 0.0000 0.5199 0.0000 nan nan

18 21 Icfu-HAA Eivi-HAA 8.0000 2.0000 8.0000 22.0000 1.0000 0.0909 nan 0.0969 nan 11.0000

18 22 Icfu-HAA Alal-HAA 6.0000 4.0000 8.0000 22.0000 0.7500 0.1818 nan 0.2082 nan 4.1250

18 23 Icfu-HAA Clha-HAA 8.0000 2.0000 8.3333 21.6667 0.9600 0.0923 nan 0.0985 nan 10.4000

18 24 Icfu-HAA Teil-HAA 7.0000 1.0000 8.1667 21.8333 0.8571 0.0458 nan 0.0473 nan 18.7143

18 25 Icfu-HAA Sapi-HAA 7.0000 3.0000 8.3333 21.6667 0.8400 0.1385 nan 0.1531 nan 6.0667

18 26 Icfu-HAA Anan-HAA 7.0000 1.0000 7.8333 22.1667 0.8936 0.0451 nan 0.0465 nan 19.8085

19 20 Sime-HAA Sias-HAA 2.0000 0.0000 8.0000 22.0000 0.2500 0.0000 0.3041 0.0000 nan nan

19 21 Sime-HAA Eivi-HAA 5.0000 2.0000 8.0000 22.0000 0.6250 0.0909 1.3438 0.0969 13.8668 6.8750

19 22 Sime-HAA Alal-HAA 8.0000 4.0000 8.0000 22.0000 1.0000 0.1818 nan 0.2082 nan 5.5000

19 23 Sime-HAA Clha-HAA 9.0000 2.0000 8.3333 21.6667 1.0800 0.0923 nan 0.0985 nan 11.7000

19 24 Sime-HAA Teil-HAA 7.0000 1.0000 8.1667 21.8333 0.8571 0.0458 nan 0.0473 nan 18.7143

19 25 Sime-HAA Sapi-HAA 9.0000 3.0000 8.3333 21.6667 1.0800 0.1385 nan 0.1531 nan 7.8000

19 26 Sime-HAA Anan-HAA 5.0000 1.0000 7.8333 22.1667 0.6383 0.0451 1.4282 0.0465 30.6960 14.1489

20 21 Sias-HAA Eivi-HAA 5.0000 2.0000 8.0000 22.0000 0.6250 0.0909 1.3438 0.0969 13.8668 6.8750

20 22 Sias-HAA Alal-HAA 7.0000 4.0000 8.0000 22.0000 0.8750 0.1818 nan 0.2082 nan 4.8125

20 23 Sias-HAA Clha-HAA 9.0000 2.0000 8.3333 21.6667 1.0800 0.0923 nan 0.0985 nan 11.7000

20 24 Sias-HAA Teil-HAA 8.0000 1.0000 8.1667 21.8333 0.9796 0.0458 nan 0.0473 nan 21.3878

20 25 Sias-HAA Sapi-HAA 8.0000 3.0000 8.3333 21.6667 0.9600 0.1385 nan 0.1531 nan 6.9333

20 26 Sias-HAA Anan-HAA 5.0000 1.0000 7.8333 22.1667 0.6383 0.0451 1.4282 0.0465 30.6960 14.1489

21 22 Eivi-HAA Alal-HAA 5.0000 3.0000 8.0000 22.0000 0.6250 0.1364 1.3438 0.1505 8.9289 4.5833

21 23 Eivi-HAA Clha-HAA 6.0000 1.0000 8.3333 21.6667 0.7200 0.0462 2.4142 0.0476 50.6803 15.6000

21 24 Eivi-HAA Teil-HAA 6.0000 0.0000 8.1667 21.8333 0.7347 0.0000 2.9189 0.0000 nan nan

21 25 Eivi-HAA Sapi-HAA 5.0000 2.0000 8.3333 21.6667 0.6000 0.0923 1.2071 0.0985 12.2544 6.5000

21 26 Eivi-HAA Anan-HAA 4.0000 1.0000 7.8333 22.1667 0.5106 0.0451 0.8566 0.0465 18.4104 11.3191

22 23 Alal-HAA Clha-HAA 1.0000 2.0000 8.3333 21.6667 0.1200 0.0923 0.1308 0.0985 1.3275 1.3000

22 24 Alal-HAA Teil-HAA 2.0000 3.0000 8.1667 21.8333 0.2449 0.1374 0.2965 0.1518 1.9534 1.7823

22 25 Alal-HAA Sapi-HAA 0.0000 3.0000 8.3333 21.6667 0.0000 0.1385 0.0000 0.1531 nan nan

22 26 Alal-HAA Anan-HAA 5.0000 3.0000 7.8333 22.1667 0.6383 0.1353 1.4282 0.1493 9.5690 4.7163

23 24 Clha-HAA Teil-HAA 2.0000 1.0000 8.5000 21.5000 0.2353 0.0465 0.2824 0.0480 5.8805 5.0588

23 25 Clha-HAA Sapi-HAA 1.0000 1.0000 8.6667 21.3333 0.1154 0.0469 0.1253 0.0484 2.5884 2.4615

23 26 Clha-HAA Anan-HAA 7.0000 1.0000 8.1667 21.8333 0.8571 0.0458 nan 0.0473 nan 18.7143

24 25 Teil-HAA Sapi-HAA 2.0000 2.0000 8.5000 21.5000 0.2353 0.0930 0.2824 0.0993 2.8430 2.5294

24 26 Teil-HAA Anan-HAA 8.0000 0.0000 8.0000 22.0000 1.0000 0.0000 nan 0.0000 nan nan

25 26 Sapi-HAA Anan-HAA 6.0000 2.0000 8.1667 21.8333 0.7347 0.0916 2.9189 0.0977 29.8761 8.0204

(C) *Analysis of synonymous versus non-synonymous nucleotide substitution rates in HAA cytoplasmic tail motif encoding fragments of Characiformes/Siluriformes/Gymnotiformes by SNAP v2.1.1 software*

The *HAA* sequence fragments of Characiformes/Siluriformes/Gymnotiformes shown in (A) were analyzed by the Synonymous Non-synonymous Analysis Program, SNAP v2.1.1, as described in (B).

Summary of Results

*Averages of all pairwise comparisons:*

ds = 1.0872 dn = 0.0577 ds/dn = 20.0296 ps/pn = 10.3316

*Averages of the first sequence compared to others:*

ds = 0.5360 dn = 0.0465 ds/dn = 11.5201 ps/pn = 8.4894

*Statistics:*

Average ps: 0.570685; Variance (ps): 0.012581; Std Deviation(ps): 0.112167;

Average pn: 0.037155; Variance(pn): 0.000662; Std Deviation(pn): 0.025730;

Average dn: 0.038769; Variance(dn): 0.160720; Std Deviation(dn): 0.400899

(C continued)

Detailed Results

Compare Sequence names Sd Sn S N ps pn ds dn ds/dn ps/pn

0 1 Asme-HAA Pime-HAA 1.0000 0.0000 7.6667 22.3333 0.1304 0.0000 0.1433 0.0000 nan nan

0 2 Asme-HAA Coma-HAA 2.0000 0.0000 7.6667 22.3333 0.2609 0.0000 0.3206 0.0000 nan nan

0 3 Asme-HAA Icpu-HAA 6.0000 1.0000 7.8333 22.1667 0.7660 0.0451 nan 0.0465 nan 16.9787

0 4 Asme-HAA Icfu-HAA 6.0000 1.0000 7.8333 22.1667 0.7660 0.0451 nan 0.0465 nan 16.9787

0 5 Asme-HAA Sime-HAA 7.0000 1.0000 7.8333 22.1667 0.8936 0.0451 nan 0.0465 nan 19.8085

0 6 Asme-HAA Sias-HAA 7.0000 1.0000 7.8333 22.1667 0.8936 0.0451 nan 0.0465 nan 19.8085

0 7 Asme-HAA Eivi-HAA 3.0000 1.0000 7.8333 22.1667 0.3830 0.0451 0.5360 0.0465 11.5201 8.4894

1 2 Pime-HAA Coma-HAA 1.0000 0.0000 7.6667 22.3333 0.1304 0.0000 0.1433 0.0000 nan nan

1 3 Pime-HAA Icpu-HAA 5.0000 1.0000 7.8333 22.1667 0.6383 0.0451 1.4282 0.0465 30.6960 14.1489

1 4 Pime-HAA Icfu-HAA 5.0000 1.0000 7.8333 22.1667 0.6383 0.0451 1.4282 0.0465 30.6960 14.1489

1 5 Pime-HAA Sime-HAA 6.0000 1.0000 7.8333 22.1667 0.7660 0.0451 nan 0.0465 nan 16.9787

1 6 Pime-HAA Sias-HAA 6.0000 1.0000 7.8333 22.1667 0.7660 0.0451 nan 0.0465 nan 16.9787

1 7 Pime-HAA Eivi-HAA 3.0000 1.0000 7.8333 22.1667 0.3830 0.0451 0.5360 0.0465 11.5201 8.4894

2 3 Coma-HAA Icpu-HAA 6.0000 1.0000 7.8333 22.1667 0.7660 0.0451 nan 0.0465 nan 16.9787

2 4 Coma-HAA Icfu-HAA 6.0000 1.0000 7.8333 22.1667 0.7660 0.0451 nan 0.0465 nan 16.9787

2 5 Coma-HAA Sime-HAA 5.0000 1.0000 7.8333 22.1667 0.6383 0.0451 1.4282 0.0465 30.6960 14.1489

2 6 Coma-HAA Sias-HAA 5.0000 1.0000 7.8333 22.1667 0.6383 0.0451 1.4282 0.0465 30.6960 14.1489

2 7 Coma-HAA Eivi-HAA 2.0000 1.0000 7.8333 22.1667 0.2553 0.0451 0.3121 0.0465 6.7084 5.6596

3 4 Icpu-HAA Icfu-HAA 0.0000 0.0000 8.0000 22.0000 0.0000 0.0000 0.0000 0.0000 nan nan

3 5 Icpu-HAA Sime-HAA 5.0000 0.0000 8.0000 22.0000 0.6250 0.0000 1.3438 0.0000 nan nan

3 6 Icpu-HAA Sias-HAA 3.0000 0.0000 8.0000 22.0000 0.3750 0.0000 0.5199 0.0000 nan nan

3 7 Icpu-HAA Eivi-HAA 8.0000 2.0000 8.0000 22.0000 1.0000 0.0909 nan 0.0969 nan 11.0000

4 5 Icfu-HAA Sime-HAA 5.0000 0.0000 8.0000 22.0000 0.6250 0.0000 1.3438 0.0000 nan nan

4 6 Icfu-HAA Sias-HAA 3.0000 0.0000 8.0000 22.0000 0.3750 0.0000 0.5199 0.0000 nan nan

4 7 Icfu-HAA Eivi-HAA 8.0000 2.0000 8.0000 22.0000 1.0000 0.0909 nan 0.0969 nan 11.0000

5 6 Sime-HAA Sias-HAA 2.0000 0.0000 8.0000 22.0000 0.2500 0.0000 0.3041 0.0000 nan nan

5 7 Sime-HAA Eivi-HAA 5.0000 2.0000 8.0000 22.0000 0.6250 0.0909 1.3438 0.0969 13.8668 6.8750

6 7 Sias-HAA Eivi-HAA 5.0000 2.0000 8.0000 22.0000 0.6250 0.0909 1.3438 0.0969 13.8668 6.8750

(D) *Analysis of synonymous versus non-synonymous nucleotide substitution rates in Characiform HAA full-length available sequences by SNAP v2.1.1 software*

The *HAA* sequence fragments of Characiformes/Siluriformes/Gymnotiformes shown in (A) were analyzed by the Synonymous Non-synonymous Analysis Program, SNAP v2.1.1, as described in (B).

Summary of Results

*Averages of all pairwise comparisons:*

ds = 0.7952 dn = 0.3739 ds/dn = 2.0202 ps/pn = 1.5883

*Averages of the first sequence compared to others:*

ds = 1.1148 dn = 0.5153 ds/dn = 2.1742 ps/pn = 1.5616

*Statistics:*

Average ps: 0.433766; Variance (ps): 0.001552; Std Deviation(ps): 0.039391;

Average ds: 0.795179; Variance ds: 0.026220; Std Deviation(ds): 0.161927;

Average pn: 0.276834; Variance(pn): 0.000476; Std Deviation(pn): 0.021825;

Average dn: 0.373858; Variance(dn): 0.007856; Std Deviation(dn): 0.088634

Detailed Results

Compare Sequence names Sd Sn S N ps pn ds dn ds/dn ps/pn

0 1 Asme-HAA Pime-HAA 63.6667 137.3333 110.8333 354.1667 0.5744 0.3878 1.0891 0.5458 1.9952 1.4814

0 2 Asme-HAA Coma-HAA 59.0000 114.0000 100.6667 319.3333 0.5861 0.3570 1.1406 0.4847 2.3532 1.6417

1 2 Pime-HAA Coma-HAA 12.8333 27.1667 91.1667 316.8333 0.1408 0.0857 0.1559 0.0911 1.7122 1.6417
